# Use of *Camelina sativa* and By-Products in Diets for Dairy Cows: A Review

**DOI:** 10.3390/ani12091082

**Published:** 2022-04-22

**Authors:** Roshan Riaz, Ibrar Ahmed, Ozge Sizmaz, Umair Ahsan

**Affiliations:** 1 Department of Animal Nutrition and Nutritional Diseases, Faculty of Veterinary Medicine, Ankara University, Ankara 06110, Turkey; roshansahil04@gmail.com; 2 Department of Animal Nutrition and Nutritional Diseases, Faculty of Veterinary Medicine, Selçuk University, Konya 42031, Turkey; vetrao6@gmail.com; 3 Department of Plant and Animal Production, Burdur Vocational School Food, Agriculture and Livestock, Burdur Mehmet Akif Ersoy University, İstiklal Campus, Burdur 15030, Turkey; uahsan@mehmetakif.edu.tr; 4 Center for Agriculture, Livestock and Food Research, Burdur Mehmet Akif Ersoy University, İstiklal Campus, Burdur 15030, Turkey

**Keywords:** *Camelina sativa*, dairy cow, milk composition, glucosinolate

## Abstract

**Simple Summary:**

*Camelina sativa*, also known as false flax, is an oilseed adaptable to wide agronomic conditions and is an environment-friendly crop utilized by human beings for decades. Camelina seed and its derivatives have a nutritional value comparable to other products fed to dairy cows. However, similar to other oil crops, it has anti-nutritional factors which have brought some regulations from authorities on the inclusion levels of camelina seed and oil-extracted products. Many studies have been conducted, and effects on feed intake, digestion, and metabolism are controversial at higher inclusion levels, Yet, there has still been no effect noticed on the metabolic hormones of dairy cows when included at an appropriate level (2 kg/animal/day seeds or 10% inclusion on a dry matter basis of oil extracted products). Although inclusion of the seed and expeller on milk fat suppression is prominent, milk fat produced by a diet including camelina seed and its by-products is rich in health-beneficial fatty acids. In addition, with processing, its anti-nutritional factors can be reduced, and solvent-extracted meals can be used in higher inclusions than current recommended levels, without affecting the digestion and metabolism of the animal.

**Abstract:**

*Camelina sativa*, belonging to the *Brassicaceae* family, has been grown since 4000 B.C. as an oilseed crop that is more drought- and cold-resistant. Increased demand for its oil, meal, and other derivatives has increased researchers’ interest in this crop. Its anti-nutritional factors can be reduced by solvent, enzyme and heat treatments, and genetic engineering. Inclusion of camelina by-products increases branched-chain volatile fatty acids, decreases neutral detergent fiber digestibility, has no effect on acid detergent fiber digestibility, and lowers acetate levels in dairy cows. Feeding camelina meal reduces ruminal methane, an environmental benefit of using camelina by-products in ruminant diets. The addition of camelina to dairy cow diets decreases ruminal cellulolytic bacteria and bio-hydrogenation. This reduced bio-hydrogenation results in an increase in desirable fatty acids and a decrease in saturated fatty acids in milk obtained from cows fed diets with camelina seeds or its by-products. Studies suggest that by-products of *C. sativa* can be used safely in dairy cows at appropriate inclusion levels. However, suppression in fat milk percentage and an increase in trans fatty acid isomers should be considered when increasing the inclusion rate of camelina by-products, due to health concerns.

## 1. Introduction

Steady increase in world human population has raised the challenge of food security for human beings. This challenge has attracted several critiques directed at the ruminant production industry, due to competition of feed supplies utilized as human food. This criticism has increased over the last few decades because of rising interest in biofuel crop production. Ruminants have a lower feed conversion ratio than monogastric animals, and their higher environmental footprint also gives cause for concern. However, ruminants can convert low-quality non-human food resources or agricultural byproducts into high-quality milk and meat [1,2]. This ability of ruminants can be enhanced further with the utilization of no-food or agro-industrial byproducts. In many countries, forages are low in protein content, and resourcing the protein is an expensive task as the countries are forced to import soybean or other protein-rich resources. To overcome these discrepancies, research for alternative feed resources has gained attention. The utilization of alternative feed resources depends on the nutritional profile, animal response to the feed, cost, and environmental impact [2,3].

*Camelina sativa* (L.) Crantz, an oilseed plant, has gained more popularity in the last decade due to its characteristic features in terms of environmental adaptability and comparable nutritional profile to conventionally used oilseeds. It can be grown in drought and dry-land/rain-fed conditions and shows more resistance to pests, as well as to other diseases [4]. Nutritionally, expeller extracted camelina cake and solvent extracted meal are rich in protein [5]. In addition, the oil content in the seed or cake contains lipids with a high amount of long-chain polyunsaturated fatty acids (PUFA), including n-3 and n-6 PUFA. Mechanical and solvent extraction reduces the crude fat and increases the crude protein and amino acid content. The lipid and fiber act as energy sources for high-producing animals. Production of methane during digestion of camelina seed and its derivatives remains low. Consequently, all these characteristics make camelina seed and its derivatives a potential animal feedstock.

Unlike other oilseed plants, camelina also contains glucosinolates, erucic acid, sinapine, and tannins as anti-nutritional factors and glucosinolates are of major concern [6]. In addition, a higher amount of camelina seed and its derivatives in the feed may contribute to a bitter taste that modulates the dry matter intake and digestion, resulting in lower milk production and poor milk composition. Previous studies, even with 20% inclusion of camelina meal, resulted in no effect on thyroid hormone production and functioning, as well as metabolism of the feed [2,7,8,9]. However, the effect of camelina seed and derivatives on feed intake, rumen digestion, and fermentation is contrasting. Similarly, the effect of camelina seeds and their derivatives on milk production and composition remain unclear, due to contrasting results [2,7,10,11].

The present review aimed to provide an account of the data and available literature on the nutritional composition of camelina seed and its derivatives, and their usage in dairy cow feeding, considering their effect on feed intake, digestion, metabolism, and milk production and composition.

## 2. Nutritional Value of *Camelina sativa* and By-Products

### 2.1. Chemical Composition

*C. sativa*, sometimes known as fake/false flax, is a flowering oilseed plant of the Brassicaceae family, generally farmed in Europe and North America. It has shown promising characteristics to be considered as a candidate as an animal feed ingredient [4]. All *C. sativa* genotypes show strong emergence and uniformly stand established after sowing. Similarly, all *C. sativa* genotypes attain flowering and seed ripening phases at 53 and 116 days after sowing, respectively; which indicates a quick seed-to-seed cycle [12].

The nutrient composition, like moisture, dry matter (DM), crude protein (CP), energy (gross, digestible, net energy for lactation), ether extract (EE), acid detergent fiber (ADF), and neutral detergent fiber (NDF), of camelina seed and derivatives is given in Table 1. *C. sativa* is a nutritionally-rich oilseed plant having DM content between 88 and 94% [2,9]. The crude protein (CP) content of CS forage is 11.34% [13], while that of camelina meal ranges from 26.5 to 41.1% [7,14], of camelina seed from 27 to 34% [7,15], and of camelina cake from 38 to 42% [16,17]. Ether extract (EE) concentration ranges between 11 and 13% [18,19]. The net energy for lactation (NE_L_) has been reported as 2.02 Mcal/kg for camelina meal, whereas it is 2.58 Mcal/kg for camelina seed [7]. The digestible energy (DE) for solvent-extracted camelina meal is 2.172 Mcal/kg [20]. The gross energy (GE) of camelina meal, camelina expeller, and cold-press camelina cake is 5.429 Mcal/kg [14], 5.139 Mcal/kg [18], and 5.057–5.197 Mcal/kg [17,21], respectively. Metabolizable energy (ME) of camelina cake is 8.0 MJ/kg for poultry, 14.0 MJ/kg for pigs, and 15.0 MJ/kg for cows [12]. The EE content of camelina expeller is 18% [22] and that of cold-press camelina cake may vary between 10.52 and 12.70% [17,21]. Crude fiber (CF) content of camelina cake ranges from 12 to 16.92% [19,21], whereas camelina forage has 21.65% CF [13]. The ash content of camelina seed has been reported between 3.7 and 6.9% [23,24]. The acid detergent fiber (ADF) ranges between 14.4% and 25.4% [7,20], whereas neutral detergent fiber (NDF) ranges between 19.8 and 49.5% [9,13]. The starch content varies between 0.2 and 1.39% [8,19]. Oil production from camelina seeds may vary from 32 to 38% [12]. The exploitation of camelina seed by-products is a crucial aspect in lowering costs and promoting environmental sustainability. The CS meal may prove to be an excellent addition to diets for ruminants attributed to these nutritional qualities.

Camelina is a rich source of minerals. Camelina seed contains 1% calcium, 1.4% phosphorus, and 1.6% potassium [27]. Potassium is the most common mineral in camelina meal, followed by sulfur, phosphorus, magnesium, and calcium [28]. Mineral profile of camelina cake, seed, and meal is given in Table 2.

### 2.2. Fatty Acid Composition

*C. sativa* is an oil plant that has received business and agriculture’s attention due to its nutritional and chemical properties [29]. Camelina seed contains 0.1 to 0.9% myristic acid (C14:0) [30,31], 5.1 to 10.3% palmitic acid (C16:0) [7,32], and 2.19 to 2.8% Stearic acid (C18:0) [11,33]. Camelina meal possesses 0.17% myristic acid (C14:0) [34], 7.19 to 9.12% palmitic acid (C16:0) [14,34], and 2.27 to 2.9% Stearic acid (C18:0) [12,31]. Camelina cake consists of 0.1 to 0.2% myristic acid (C14:0) [30,32] and 7.19 to 9.46% palmitic acid (C16:0) [19]. Camelina seed has exhibited 14.4 to 19.9% oleic acid (C18:1) [7,30], 13.5 to 28.5% linoleic acid, (C18:2) [7,35], and 28.6 to 36.77% linolenic acid, (C18:3) [11,32]. Camelina seed contains 14.4 to 19.9% oleic acid (C18:1) [7,30], 13.5 to 28.5% linoleic acid, (C18:2) [7,35], and 28.6 to 36.77% linolenic acid, (C18:3) [11,32]. Camelina meal contains 17.8 to 21.7% oleic acid (C18:1) [7,36], 24.35 to 28.8% linoleic acid, (C18:2) [31,36], and 24.2 to 46.3% linolenic acid, (C18:3) [7,36]. The camelina seed oil is one of the richest known plant sources of the n-3 PUFA especially alpha-linolenic acid (ALA) [29,37]. Camelina seed contains 55.2–57.1% of PUFA [25,38], 9.04–13.3% of SFA [31,39], and 32.1–36.16% of mono unsaturated fatty acids (MUFA) [31,38]. Camelina oil (CO) has PUFA (55.2%), SFA (10.2%) and MUFA (34.6%) [40]. Table 3 represents the fatty acid (FA) composition of camelina seed, camelina cake, and camelina meal.

The oil content of camelina seed has been found to range between 300 and 490 g kg^−1^ [41,42]. Camelina oil is rich in oleic, (18:1, 14 to 16%), linoleic (LA), (18:2, 15 to 23%), alpha-linolenic (ALA), (18:3, 31 to 40%), and eicosenoic (20:1, 12 to 15%) acids. Other minor fatty acids include palmitic (16:0), stearic (18:0), and erucic (22:1) acid [43,44]. Camelina seed is high in anti-oxidants like phenolic acids and flavonoids, tocopherols, and xanthophyll, in addition to PUFAs and proteins [25,45]. Gamma tocopherol makes for over 90% of the total tocopherols [37]. These chemicals have an antioxidant effect, as well as an influence on the flavor and color of the oil [45]. Antioxidants stabilize oils and prevent unsaturated fatty acids from oxidation, thus prolonging the shelf life of camelina oil for up to six months [37]. From a nutritional perspective, the fatty acid composition appears to be quite important; linolenic acid and alpha linolenic acid are important fatty acids, and the oil can improve the n-6/n-3 balance in a diet, increasing the biological value of the food [46]. During seed development, the amount of linolenic acid in oilseeds fluctuates with temperature. ALA synthesis diminishes at higher temperatures, increasing the two other primary ingredients, oleic and linoleic acids [47].

### 2.3. Amino Acid Composition

Camelina seeds contain at least 17 amino acids. The primary constituents of essential amino acids are leucine, valine, lysine, phenylalanine, and isoleucine. Besides essential amino acids, protein in camelina seeds is high in non-essential amino acids such, as glutamic and aspartic acids, serine, proline, and arginine [60,61]. The most abundant essential amino acid available in camelina seed is arginine [62]. Camelina meal contains a high content of crude protein and essential amino acids that make it a viable protein and energy source for ruminants and non-ruminants alike [5]. Camelina meal is obtained from camelina seeds after oil extraction [21]. The amino acid profile of camelina seed, cake and meal are given in Table 4.

## 3. Factors Governing the Nutritional Value of Camelina Seed and Camelina By-Products

Nutritional composition and yield of camelina seed and its derivatives vary based on variety (winter, fall, summer, and spring), genotype (Lindo, Ukrajinskaja, Soledo, Volynskaja, ZarjaSocialisma, and Bavaria), environmental circumstances (temperature, humidity), and agronomic practices (planting timing, fertilizer use, soil condition, irrigation or non-irrigation of the soil, and herbicide use).

Winter *C. sativa* has a larger root to shoot ratio than spring camelina, probably due to a prolonged period in fall for roots to establish themselves before overwintering and beginning of development the following spring, resulting in more root growth [51]. Cultivars, climate, and soil types where camelina is grown all influence the seed yields [41]. In Mediterranean conditions, the highest seed yields have been documented [63].

Seeds of *C. sativa* are quite tiny (0.7 mm to 1.5 mm), with a 1000-seed weight ranging from 0.8 to 1.8 g, depending on cultivar and growth circumstances [64]. Despite its modest genetic diversity, modern camelina germplasm contains enough phenotypic variety to allow for significant agronomic advancement [61]. Oil content of camelina seed is a highly heritable feature [65] and seed yield appears to be positively associated with genotype [66]. Amplified fragment length polymorphism fingerprinting indicated a significant degree of genetic variation in a hitherto inaccessible group of *C. sativa* accessions from the Russian-Ukrainian region [67]. Dry matter content and oil content of camelina is mainly dependent on the number of days after anthesis [68]. Similarly, accumulation of protein and oil content in *C. sativa* seed is also dependent on the number of days after anthesis [69].

Although camelina may be effectively grown in semi-arid conditions [70], heat stress can be an issue, particularly during the reproductive period. When high temperatures coincide with the reproductive phase, camelina seed yields and oil content decrease dramatically, despite ample availability of water [71]. In camelina, protein content of seed is favorably connected with nitrogen fertilizer rate, whereas seed oil content has been negatively correlated [72]. The nature of the oil and the amount of protein in the seed appear to be substantially determined by genotype. Availability of minerals in soil (Sulfur) greatly influences PUFA concentration in *C. sativa* seed [73]. Camelina should be harvested when 75 percent of the silicles are ripe, according to Sintim et al. [74], to establish a balance between seed output, seed oil content, and tolerable loss due to breaking. To produce a high-quality seed, post-harvest seed washing and conditioning are required.

Major factors affecting the nutritional composition of camelina seed and its derivatives are discussed below.

### 3.1. Variety

Different varieties of *C. sativa* contain distinct amounts of protein and oil content [75]. The summer variety contains more oil and protein content as compared with the winter variety [76]. Summer camelina seeds contain 40.9% oil content, whereas winter camelina seeds have 38.9% [75]. The amounts of SFA and MUFA are higher and of PUFA lower in summer camelina [76]. The summer variety requires less amount of fertilizer or pesticide and has more tolerance for draught and cold climates [77]. There are differences in summer and winter biotypes in regards to vegetative leaf pairs, breadth, length, height, and lobe numbers [78]. Schuster and Friedt et al. [79] stated that summer camelina varieties contain a higher content of glucosinolate.

### 3.2. Genotype

The major concern about camelina in animal feed is the anti-nutritional factors, which greatly vary with genotype. For instance, *Ames28371* and *FF006* contain the lowest amount of glucosinolate, *Calena* contains the lowest amount of phytic acid, *D11851* contains the lowest amount of sinapine, whereas *Ligena* and *D9952* contain the lowest amounts of condensed tannins [80]. Colombini et al. [12] compared 10 genotypes of *C. sativa* and concluded that the highest yield per hectare, CP, plant height and weight, and number of branches and siliques of the main stem were obtained by *CAM40*, *FF066, ligena* and *FF084*, respectively. Twelve genotypes of *C. sativa* were evaluated by Katar et al. [56], mainly focusing on linoleic, erucic, oleic, linolenic, eicosenoic, and stearic acids. The latter author stated that *Ames28372* can be used as an oil crop due to its medicinal, industrial and biodiesel importance. Jiang et al. [73] studied 5 genotypes of *C. sativa* and stated that *CD1007*, *CD1002* and *CD1005* contain the highest amounts of oil and protein yields and PUFAs, protein content, and MUFAs, respectively. A variety called *dikiy* found in Crimea can produce a high yield not only under a favorable environment, but also under adverse and stressful situations [81].

### 3.3. Environment

Different environmental factors like composition of soil, precipitation and surrounding temperature greatly influence the oil quality, and quantity of *C. sativa*. The oilseed content concentration is dependent on temperature [73]. High temperature leads to reduced oil concentration [82]. Precipitation and temperature influence the levels of phospholipids, tocopherols, and phytosterols in *C. sativa* [82]. Raziei et al. [83] cultivated *C. sativa* in cold and tropical provinces and measured the following characteristics of *C. sativa*: SFA, MUFA and PUFA. The latter authors found that the proportion of unsaturated FA was greater in cold regions. Similarly, Obour et al. [84] stated that *C. sativa* grown in the Northern Great Plains, having cold temperatures, have superior fatty acid profiles as compared with those grown in the Central Great Plains. In summary, *C. sativa* should be cultivated in cold areas for nutritional usage, and in tropical areas for industrial usage.

### 3.4. Agronomic Practices

Production of *C. sativa* increases with nitrogen fertilization usage. The yields of summer and winter varieties improved by 2.3 and 3.7 times with 90 and 100 kg per hectare nitrogen fertilization [75]. Increase in *C. sativa* seed protein content and decrease in oil content as nitrogen fertilizer levels increases as mentioned in studies [85,86]. The production of AA and FA is widely considered to compete for energy metabolism and carbon skeletons [66]. Since seed yield is positively related to N rates, oil and protein yield increased with N input. The concentration of PUFA enhances as N increases, but the concentration of MUFA decreases [73]. Ahmad et al. [87] cultivated *C. sativa* with the application of selenium in the form of prime and foliar, and concluded that Se triggers several physio-biochemical properties during drought stress and mitigates the negative consequences of drought stress. Kirkhus et al. [82] showed that seed oil content and composition of *C. sativa* are considerably influenced by pre-crop, S and N fertilizer levels and season. Nitrogen fertilization reduced oil content while increasing the amounts of tocopherols and 18:3 in the camelina oil.

## 4. Comparison of Camelina Meal with other Oilseed Meals

Camelina is more drought- and cold-resistant than canola [88], therefore, camelina production is expected to rise [89]. Camelina meal has higher CP (39.5%) and NDF (37.5%) values than expeller-extracted camelina meal; however, a lower EE (1.3%) has been reported. Camelina meal possesses a lower crude protein (CP) content (39.5%) than soybean meal 44 (46.7%). Nevertheless, the CP content is equivalent to sunflower meal 36 (39.3%) and rapeseed meal (39.8%) [90]. A major portion of camelina meal consists of glutelin (64.64%) followed by globulin (17.67%), and albumin (10.54%) [91]. Protein yields for cold-pressed defatted camelina meal and hot-pressed defatted camelina meal have been reported as 38.4% and 36.8%, respectively [92]. Berti et al. [51] demonstrated that protein yield of camelina meal is greater by solvent extraction using hexane (35.9%) in comparison with cold-press (29.9%) and CO_2_ extraction (31.6%). An upward trend has been seen regarding the use of cold-press camelina cake (CPCC) in animal diets over the years [16,93]. Researchers have estimated that CPCC is comprised of 30% CP and 10 to 30% oil that contains 35% α-linolenic acid (ALA) [93]. Kahindi et al. [16] found that CPCC, like canola byproducts, has a high EE content, which is attributed to seed varity and coat thickness along with its starch percentage. Camelina meal contains about 15% crude fiber (CF) mostly accounted for by cellulose. The most EE content was in expeller-extracted camelina meal (CE) (13.5%) [90].

Camelina hulls exhibit equal NDF (54.9%) and ADF (34.8%) content, and have higher ADL (7.8%), and lower CP (9%) than soybean hulls (17.1%) [90]. The NDF of camelina seed cake (CSC) is greater than that of canola and solvent-extracted soybean meal, which is 32–34%, 22.6%, and 8.21% respectively [21,94]. Camelina meal could be a good alternative to soybean meal and maize in diets for growing heifers [26]. In dairy cows, a study of raw, moist and dry-heated camelina seeds revealed that moist heat was the most effective treatment for lowering the rumen-degradable protein (RDP) and enhancing the digestibility of rumen-undegradable protein (RUP) [15].

Considering the above-mentioned comparisons, it can be concluded that partial or complete replacement of major oilseed meals, like soybean, sunflower, rapeseed or cottonseed meals, with camelina meal is possible. The replacement of major oilseed meals with camelina meal will not only ensure self-reliance in oilseed meal, but also improve profitability in addition to reducing the import bill of oilseeds.

Ruminants benefit from camelina meal and seeds since they are high in protein and energy [95]. The researchers observed that camelina oil from the cold-press technique had low oxidative stability which was linked to a high PUFA concentration (57.1 to 76.4%). However, camelina oil was more stable than cold-press linseed oil [96,97], thus camelina oil can improve the fatty acid composition of milk and meat [98]. Indeed, 72% organic matter (OM) and 77% CP digestibilities were discovered in an in vivo study which were comparable to sunflower and maize gluten meal [99]. The cheese produced from milk of cows fed a camelina meal-based diet rich in ALA had greater conjugated linoleic acid (CLA) compared to those fed linseed, soybean, or rapeseed meals [100]. A brief comparison of nutritional contents of camelina meal with other oilseed meals has been presented in Table 5.

## 5. Anti-Nutritional Factors in Camelina Seed and By-Products

Plants tend to synthesize anti-nutritional factors (ANFs) in the form of secondary metabolites that safeguard them against insects, pathogens (bacterial or viral), predators (omnivorous or herbivorous), and negative environmental alterations [107]. The ANFs of camelina seed, cake, and meal are given in Table 6. Camelina seeds and roots contain the majority of ANFs [6]. A previous study found that the levels of phytic acid, condensed tannins, and sinapine in camelina meal are lower than those found in other members of the *Brassicaceae* family commonly used in animal diets [12]. A few studies have evaluated the trypsin inhibitor action in camelina. A study reported trypsin inhibitor activity between 16 and 21 units per milligram on a dry weight basis [33], which is high enough to raise some concerns. Heat treatment, on the other hand, may diminish activity, and there is enough variability to suggest that selective cultivar breeding may be able to lower it.

Camelina meal, despite being a high-quality meal with an amino acid profile similar to soybean meal [109], contains a variety of ANFs [110]. Due to ANFs such as glucosinolates, tannins, phytic acid, fiber, and trypsin inhibitors, inclusion of camelina seed and its by-products is typically confined to low levels [16]. Oil seeds from the *Brassicaceae* family, including canola, mustard, and rapeseed, contain glucosinolates [111]. Camelina contains glucosinolates (14–36 μmol/g), at a level similar to that observed in rapeseed but at much lower levels than that found in other crops like mustard (>120 μmol/g) [18,35]. Camelina contains a unique glucosinolate, 10-methyl-sulfinyl-decyl glucosinolate (10-MSG) that accounts for 60 to 65% of the glucosinolates found in camelina, while 9-methyl-sulfinyl-nonyl glucosinolate (9-MSG) and 11-methyl-sulfinyl-undecyl glucosinolate (11-MSG) are approximately 30% and 10%, respectively [79,110].

Winter camelina cultivars have roughly 10 mol/g fewer glucosinolates than frequently planted spring types, and glucosinolate content is mostly dependent on sulfur content and soil type [79]. The main issue while feeding camelina is glucosinolate because of its effects on the thyroid and cardiovascular systems [112].

Camelina has a sinapine concentration of about 4 mg/g seed, which is about 30% lower than rapeseed [110]. Inositol pentaphosphate and inositol hexaphosphate values range from 20 to 22 mg/g, the highest amounts being seen in winter cultivars, and also vary according to the region where they are grown [110].

Condensed tannins are found in all seeds and may reduce digestion in ruminants and non-ruminants alike [110]. The tannin level in camelina is 1.1 mg/g which is much lower than 4 mg/g in rapeseed [110]. The majority of ANFs in camelina are equivalent or slightly lower than canola and rapeseed.

Camelina seed contains (~3%) erucic acid [29] that causes myocardial lipidosis in animals. The European Union has defined the maximum dietary limit of erucic acid at 7 mg/kg body weight [113]. According to Hrastar et al. [114], the erucic acid level has been minimized by extensive breeding in some crops to 0%; canola meal now contains <2% erucic acid. However, camelina still contains 2-4% of erucic acid.

## 6. Reduction of Anti-Nutritional Factors and Enhancement of Nutritional Value of *Camelina sativa*

### 6.1. By Processing

Solvent extraction [115], enzyme addition [116], and heat treatment [117] are some of the procedures that may be used to lower the ANFs in camelina.

Earlier studies have reported thermal and chemical degradation (myrosinase catalyzed hydrolysis) of glucosinolates [118,119]. Glucosinolate profiles of cooked brassica vegetables may vary depending on the culinary techniques used; such as cooking, steaming, or microwaving. During the cooking process, the indolic glucosinolate of red cabbage (*Brassica oleracea* var. *capitata* L.) was reduced [120]. According to a recent study [119] on the roasting of rapeseed, industrial-scale post-harvest treatments have an impact on the glucosinolate profile of plant materials. During the roasting process, up to 29% of the original glucosinolate amount in plant materials was reduced [23]. These findings show that thermal degradation reduces the glucosinolate content of plant materials in industrial-scale roasting processes, with up to one-third of glucosinolates degraded. Aerobic conversion of meals results in enhanced CP and EE content, and decreased glucosinolate concentration (up to 70%) in meals.

Phytates and tannins are heat-stable compounds that can be reduced by dehulling if they are stored in the outer seed coat [121] or decomposition via other methods, such as fermentation [122]. Dry or wet heating [123], as well as soaking in alkaline solutions like ammonia or calcium hydroxide [124], can reduce sinapine. To increase CP content, non-starch polysaccharides (NSPs) are fermented through microbes and release sugar for microbial consumption. Microbial conversion of fructose, galactose, glucose (hydrolytic products of oligosaccharides), and NSPs into organic acids (acetic, propionic, and butyric acids) serve as an energy source [125]. Reduction of ANFs in diet leads to increase in utilization of the plant nutritional profile at maximum level in diets.

### 6.2. By Solid-State Fermentation

Solid-state fermentation (SSF) is a bioprocessing technique that improves the nutrient composition of several underutilized agricultural byproducts and crops. Anti-nutrients are catalyzed during SSF, which includes the synthesis of exogenous enzymes along with their use as a microbial substrate for their growth [126,127]. *Aspergillus* spp. is one of the most important filamentous fungi in the food and fermentation industries, and they are generally recognized as safe [128].

Non-fermented camelina meal has a phytic acid content of 27.48 mg/g that was significantly reduced to 22.39, 16.72, and 18.98 mg/g with the help of *Aspergillus sojae*, *Aspergillus ficuum*, and co-culture fermented camelina meal, respectively [129]. Camelina meal’s phytic acid content was previously reported to be reduced during fermentation [130,131]. Lowering of pH in fermented meals activates the fungi’s phytase leading to lower phytic acid content [132] in addition to the reduction of total glucosinolates between 26.16 μmol/g and 30.35 μmol/g [129] from 34.43 μmol/g [133]. This could be due to differences in camelina meal sources and processing conditions.

### 6.3. By Genetic Engineering

*C. sativa* is a hexaploid species that has received considerable attention in the last decade because its oil traits are different from those of other oilseed crops due to its high level of n-3 PUFA, tocopherols, and ease in genetic engineering [134,135]. The first FA modification in *C. sativa* was achieved by overexpressing the fatty acid hydroxylase gene from castor (*Ricinus communis RcFAH12*) driven by seed-specific phaseolin promoter that resulted in 15% hydroxy fatty acid (HFA) production in camelina plants that could not produce it earlier [136]. Second, antisense silencing reduced the expression of the camelina fatty acid desaturase 2 (CsFAD2) enzymes, blocking the desaturation pathway and accumulating more oleic acid (18:1). The level of oleic acid (18:1) was raised from 15.5% to 51.2% while levels of linoleic acid (18:2) and linolenic acid (18:3) were reduced from 16.8% and 33.2% to 6.3% and 11%, respectively [137].

Another study found that RNAi silencing of fatty acid desaturase 2 (*FAD2*) and fatty acid elongase 1 (*FAE1*) improved the oleic acid content even more, with 18:1 accumulation reaching 70%, and 18:2 and 18:3 levels dropping from 17% and 36% to 4% and 8%, respectively [138]. The *FAD2* knock out using CRISPR/Cas9 by two different research groups in 2017 was the first target of genome editing in camelina [139,140]. These studies used gRNA to target all three *FAD2* loci at the same time and showed a significant increase in oleic acid and a decrease in PUFAs like linoleic acid and linolenic acid. Camelina seed oil contains a high level of very-long-chain fatty acids (VLCFAs) that make it unsuitable for industrial or consumer use. Use of CRISPR/Cas9 to deactivate the *FAE1* gene increased the C18 unsaturated fatty acids (such as oleic, linoleic, and a-linolenic acids) and decreased the C20–C24 VLCFAs (such as eicosenoic acid and erucic acid) to less than 2% [141].

Recently, Huang et al. [142] stated that it is possible to create *C. sativa* dihydrodipicolinate synthase (*DHDPS*) isoforms that are resistant to lysine feedback inhibition, resulting in a higher level of protein-incorporated lysine in the seed. This study also revealed that individual mutations, as well as combinations of mutations, must be examined within the context of the enzyme under investigation to generate variants that are not only lysine insensitive but also highly active. It may be possible to re-engineer one or more of the endogenous *C. sativa DHDPS* paralogues to confer these properties using the ability to edit genes directly within the *C. sativa* genome [143].

It can be concluded from the above discussion that the nutritional profile of *C. sativa*, in terms of FA and amino acids, can be enhanced with the help of genetic engineering.

## 7. Use of Camelina Seed and By-Products for Dairy Cows

### 7.1. Effects on Feed Intake and Digestion

The DM content of diet is the principal component in making the ration for animals. The DM intake (DMI) and its digestibility is an important factor in influencing the performance of dairy cows. In high-producing animals, DMI plays a key role to supply the required amount of nutrients. Therefore, attention should be paid to DMI of animals. Heifers fed diets with 10% camelina meal, linseed meal, or distiller’s dried grains with solubles (DDGS) on a DM basis show no difference in DMI and average daily gain (ADG). Although gain: feed was lower for camelina meal compared to linseed meal, it was not different in comparison with DGGS. Treatments also showed that the body condition score was greater for the camelina meal group compared to the linseed meal and DDGS groups [8]. These differences can be due to differences in digestibility and metabolism of camelina meal and linseed meal.

Effects of camelina seed or its derivatives feed on the DMI of dairy cows remain inconclusive. The addition of 630 g/d camelina seed or 2 kg/d camelina meal with corn silage-based (60%) diet had no effect on DMI [7]. Cows fed a clover silage-based diet along with expeller extracted camelina meal or camelina oil, having an equivalent amount of lipids (29 g/kg) at the inclusion rate of 20% of concentrate feed, showed no effect on silage and total DMI, OM intake (OMI), and NDF intake compared to the control diet. However, expeller extracted camelina meal lowered the OMI [2]. Sarramone et al. [11] reported no change in DMI, and OMI, CP, and NDF intakes in dairy cows fed a diet with expeller extracted camelina meal and camelina seed (Table 7). Camelina meal and expeller extracted camelina meal have higher amounts of PUFA [2], which can be the reason for lower DMI. due to influencing DM digestibility, fermentation, and shift in the digestion site to the intestine from rumen [144].

Although expeller extracted camelina meal and camelina meal tended to decrease the DMI, inclusion rates (2 kg/d, 10% DM basis, 20% inclusion in concentrate feed) did not show any significant decrease in the DMI. Therefore, further studies are recommended with higher inclusion rates and feed regimes to evaluate the selection behavior and DMI in dairy cows. Most often, lower DMI is attributed to the unsaturated FA present in the oilseeds and their derivatives [145]. Higher unsaturated FA content in the oilseeds or their derivatives can regulate the physiology of the rumen. They change microbial digestion, and site of digestion, and exert a filling effect in the rumen, resulting in lower feed intake. Although profound decrease in DMI has not been reported at the studied inclusion levels of camelina seeds and derivatives, solvent extracted camelina meal could be studied in the future with higher inclusion rates.

Halmemies-Beauchet-Filleau et al. [2] described that OM, NDF, nitrogen, and total tract digestibility in dairy cows fed a red clover silage-based diet with camelina meal or camelina oil remained unaffected. Similarly, Lawrence et al. [8] showed no effect of camelina meal on total tract digestibility in heifers; whereas OM digestibility of camelina meal was greater in comparison with linseed meal. Replacement of canola meal with solvent extracted camelina meal did not show any difference in DM, OM, ADF, CP true digestibility; however, NDF digestibility was decreased. Such differences require further investigation [9]. However, this difference in digestibility might be attributed to the effect of camelina meal on rumen microbes and fermentation process.

### 7.2. Effects on Rumen Fermentation and Rumen Microbial Population

Lawrence et al. [8] reported no difference in rumen pH, total VFAs, acetate, propionate, acetate to propionate ratio, iso-butyrate, valerate, and isovalerate in heifers offered a diet containing camelina meal. However, butyrate decreased with the supplementation of camelina meal. The NH_3_ production in the rumen by both the treatments remained unchanged. Similarly, inclusion of camelina seed in diets with two fat levels increased the C4 and C5 branched chain VFAs, propionate, and valerate, reduced the concentration of acetate and total VFAs, while not affecting formate, lactate, and succinate [146]. Hurtaud and Peyraud et al. [7] stated that no change occurs in rumen pH after feeding camelina seeds and meal compared to a control diet. Diets with camelina seed or meal decreased acetic acid, acetate to propionate ratio, and increased butyric acid and propionic acid. This increase in butyric and propionic acids was greater in the group fed camelina meal compared to camelina seeds. Iso-acids remained unchanged under the effect of the treatments. Camelina meal and camelina seeds added to the diets showed no effect on plasma glucose and urea. Camelina meal increased total glycerol and reduced plasma alpha-amino N but these components remained unchanged with camelina seeds compared to a control diet [7]. Replacement of canola meal with camelina meal decreased the acetate, and acetate: propionate, and increased the propionate, valerate, isovalerate, and branched chain VFAs, whereas, pH, total VFAs, butyrate, and iso-butyrate remained unchanged [9]. Sarramone et al. [11] also reported a decrease in acetate and iso-butyrate in addition to decrease in butyrate, propionate, acetate: propionate in response to feeding camelina seeds and camelina meal. These researchers also reported an increase in the NH_3_-N of dairy cows fed camelina meal compared to those fed DDGS. However, it was lower in comparison with those fed camelina seeds. Sizmaz et al. [3], in an in vitro study, reported no change in pH, NH_3_-N of camelina meal compared to soybean meal. However, production of total VFAs, acetate, and fermentative CH_4_ decreased, and propionate increased while acetate: propionate ratio, butyrate, isobutyrate, valerate, and isovalerate remained unaffected. Increasing levels of dietary camelina oil (0, 2, 4, 6, and 8%) in total mixed rations having 30:70, 50:50, and 70:30 roughage to concentrate ratios showed a marked decrease in CH_4_ production after 48 h of incubation and increase in ammonia nitrogen and microbial protein in 30:70 and 50:50 TMRs [39]. The study also reported that camelina oil lowered the methanogens, protozoa, bacteria, *Prevotella*, as well as increased the *Firmicutes* to *Bacteroidetes* ratio, *Pseudobutyrivibrio*, and *Ruminobacter* in camelina oil groups [39]. Similar results were reported by Dai et al. [146] in response to the inclusion of camelina seed in diets in an in vitro study. They reported an increase in *Firmicutes*, *Bacteroidetes*, *Erysipelotrichaceae*, *Succinivibrionaceae*, and *Veillonellaceae* while a reduction in relative abundance of *Butyrivibrio* spp., *Fibrobacter* spp., *Ruminococcus* spp., *Lachnospiraceae*, *Paraprevotellaceae*, *Ruminococcaceae*, and *Fibrobacteraceae*, in addition to increased abundance of *Succinivibrio* and *Megasphera* genera in groups with camelina seed [146]. Similarly, supplementation of 60 g camelina oil had a decreasing effect on ruminal CO_2_ and CH_4_ production in addition to no effect on total fungi, protozoa, bacteria, and methanogens [40]. This decrease in CH_4_ production also shows the environmental benefits of the use of camelina products in animal diets that might be attributed to the reduction of methanogens [39] and/or the bio-hydrogenation of unsaturated fatty acids capturing the hydrogen atoms necessary for CH_4_ formation in the rumen. Moreover, camelina by-products rich in oil, i.e., camelina seed, cake, and oil, being rich in PUFAs can shift the composition of ruminal microbial population; thus, inducing substantial alterations in rumen fermentation, metabolism, and metabolite composition.

Brandao et al. [9] observed an increase in branched chain VFAs in an in vitro study involving partial or complete replacement of canola meal with camelina meal. Besides these, increase in branched chain VFAs, decrease in NDF digestibility, no effect on ADF digestibility, and lowered acetate levels occur, due to the replacement of canola meal with camelina meal. These findings suggest the suppression of cellulolytic bacteria without affecting microbial efficiency in the rumen of dairy cows fed diets with camelina meal. This notion was further confirmed by Dai et al. [146] who reported a decrease in cellulolytic bacterial populations and communities in addition to those that produce acetate. In general, camelina seed and its derivatives are rich in PUFAs that play a key role in modifying the microbial population of rumen. A shift in microbial communities takes place mainly through the disruption of lipid bilayers of cellulolytic bacteria [147], thereby suppressing NDF digestibility and acetate production while enhancing production of branched chain VFAs. Microbial populations other than cellulolytic bacteria needing branched chain VFAs for growth in the rumen might be a reasonable explanation for increased rumen branched chain VFAs in dairy cows fed camelina seed and its derivatives.

### 7.3. Effects on Metabolism

In general, the rumen environment is influenced by diets composition and inclusion rate and consequent change in the microbial community and fermentation. As described above, glucosinolates present in camelina are a matter of concern for using it as a replacement of the protein and fat source(s) in ruminant diets, because of their toxic effects on thyroid functioning and metabolic imbalance. However, Lawrence et al. [8] reported no change in plasma glucose, urea N, triglycerides, cholesterol, IGF1, and T3 and T4 in heifers fed diets containing 10% camelina meal. In the same manner, diets containing camelina seed and camelina meal showed no effect on plasma NEFA (non-esterified FA), glucose, and urea. Total plasma glycerol and alpha-amino N decreased with camelina meal, while remaining unchanged with camelina seed supplementation [7]. Similarly, Halmemies-Beauchet-Filleau et al. [2] reported no change in plasma NEFA and glucose concentration in dairy cows fed expeller-extracted camelina meal and camelina oil. Likewise, diets having camelina meal did not alter the plasma levels of beta hydroxybutyrate, insulin, and T4 [26]. Similarly, feeding rations with camelina meal had no effect on the plasma SFA, MUFA, thyroid stimulating hormone, T3, T4, and cortisol. However, an increase in plasma total FA, n-3, and n-6 PUFA was noted [36]. Brandao et al. [115] reported a reduction in non-ammonia nitrogen, bacterial nitrogen, and dietary nitrogen, but increased ammonia nitrogen in groups receiving 17.7% camelina seed at 8% dietary EE compared to those with 17.7% camelina seed at 5% dietary EE. In addition, rumen undegradable protein nitrogen, rumen degradable protein nitrogen, and the pH of the fermentator remained unaffected. These findings indicate that microbes had reduced nitrogen synthesis, probably due to the toxic effect of oil content of camelina seed on microbial population. These studies suggest that the inclusion of camelina seed and by-products can be used safely in dairy cows at an appropriate inclusion rate.

### 7.4. Effects on Milk Production, Bio-Hydrogenation, and Milk Composition

Use of dietary oilseeds or their derivatives affect milk yield and composition. These effects are dependent on the inclusion levels, derivative type, concentration of unsaturated fats, and composition of the basal diet. The effect of camelina seeds and their derivatives remain unclear due to contrasting results. Earlier studies reported that milk production of dairy cows remains similar despite the inclusion of camelina seed or its derivatives in diets [2,7,10]. However, a recent study reported that dietary inclusion of expeller extracted camelina meal lowered the energy and fat corrected milk yield, and milk fat and protein yields in comparison with DDGS (Table 8) [11].

Hurtaud and Peyraud et al. [7] demonstrated that milk fat yield and FA composition of milk fat is influenced by the addition of camelina meal in diets of dairy cows compared to control and camelina seeds. Camelina seed and camelina meal reduced the SFA while an increase in MUFA, PUFA was noted in comparison with a control. Feeding camelina seed and camelina meal enhanced all *trans*-isomers of C18:1, particularly the *trans*-10 C18:1. In addition, an increase in trans FA was noted in milk of dairy cows fed diets with camelina seed or camelina meal, while short-chain MUFA i.e., C14:1 and C16:1 and medium-chain FA (4- to 12- carbon FA) were lower. Cows fed diets with camelina meal exhibited a suppression of *cis*-9 and *cis*-12 C18:1 isomers in milk. Among the PUFA, dietary camelina seed and camelina meal increased the C18:2 isomers especially CLA and rumenic acid (cis-9, trans-11 CLA). However, this response was more pronounced in cows fed camelina meal than those fed camelina seed [7]. Camelina seed and camelina meal increased the LA despite a very low level of LA in milk. In contrast, inclusion of expeller extracted camelina meal and camelina oil had no effect on milk fat yield; however, composition of milk fat was altered by camelina meal and camelina oil in terms of increased MUFA and PUFA and increased SFA compared to control diet [2]. Nonetheless, expeller extracted camelina meal had greater PUFA, MUFA, and CLA in addition to lowered SFA than for camelina oil [2]. Partial or complete replacement of sunflower meal with camelina meal did not affect the milk yield and milk composition; although MUFA, PUFA, CLA, n-3 PUFA, n-6 PUFA, α-LA, and CLA increased in milk of dairy cows fed a control diet with partial or complete replacement of sunflower meal [10]. It is well accepted that the FA yield and composition of milk can be modified through modulation of dietary FA. Dietary camelina oil or expeller-extracted camelina meal lowered the 4- to 14-carbon FA and increased the trans FA in milk. Dietary camelina oil reduced the C18:0, *trans*-4, *trans*-6 to *trans*-12 C18:1, and increased *cis*-15 C18:1 compared with sunflower oil. Moreover, dietary camelina oil suppressed the n-6 C18:2, *trans*-10, *cis*-12 CLA, and *trans*-10, *trans*-12 CLA in addition to enhancement of other isomers of C18:2, n-3 C18:3, C18:3, C20:0, and C20:1 [2]. Findings suggest that dietary camelina oil might have exerted these effects due to increased intake of n-3 C18:3. Similarly, dietary camelina oil lowered the C6:0, C8:0, C10:0, C12:0, C14:0, and C16:0 while increasing the C18:0, total C18:1, C18:2, CLA, n-3 C18:3, MUFA, PUFA, and *trans* FA levels in milk of dairy cows [40]. Bayat et al. [40] reported an increase in *cis*-10 C16:1, *cis*-12 C16:1, *trans*-9 to *trans*-13 C16:1, *cis*-9, *cis*-12, *cis*-15, *cis*-16 C18:1. Milk fat content and composition changes with the addition of camelina diets are shown in Table 9. Camelina seed is a rich source of PUFA that usually stimulates ruminal bio-hydrogenation to an extent, followed by decrease in bio-hydrogenation of FA and transportation to the intestine for absorption. Therefore, it can be said that decreased ruminal bio-hydrogenation enhances the composition of milk FA in terms of desirable FA [146]. Camelina seed and oil rich by-products of camelina seed increase the total CLA content of milk, notably rumenic acid (*cis*-9, *trans*-11 CLA). This change in the yield of fats can be explained by two facts. First, the addition of camelina seed or its derivatives in the diet results in the suppression of acetate production, which is the precursor for the FA yield in the milk. Second, during microbial fermentation, there is a shift from a *trans*-11 to *trans*-10 bio-hydrogenation pathway, converting to more intermediates and short chain fatty acids, which results in lower milk fat yield. A diet rich in PUFA has a considerable effect on the content in milk, implying greater food quality and possible health benefits for consumers. A detailed summary of the effect of camelina seed and camelina by-products on the FA composition of the milk fat of dairy cows is shown in Table 10. Addition of camelina to dairy cow diets decreases ruminal bacteria of *Pseudobutyrivibrio* and *Butyrivibrio* genera in addition to *Clostridium proteoclasticum* and cellulolytic bacterial species like *Ruminococcus albus*, *Ruminococcus flavefaciens*, and *Fibrobacter succinogenes*, thus suppressing bio-hydrogenation [146]. Suppression of bio-hydrogenation increases desirable FA transfer to milk. Suppression of 6- to 16-carbon FA and SFA levels in the milk of dairy cows fed camelina oil is attributed to the high content of oil in plant sources, including camelina oil, that ensures the availability of 18-carbon and above FA. Consequently, the availability of C18 and further long chain FAs causes the inhibition of acyl-CoA carboxylase, thus suppressing the de novo biosynthesis of 6- to 16-carbon FA in mammary glands [148,149]. Enhancement of *cis*-MUFA in the milk of dairy cows fed camelina seed and oil rich by-products is mainly due to increased intake and subsequent escape of *cis*-9 C18:1 from the rumen that undergoes desaturation in the mammary glands since most C18:0 in blood circulation is desaturated in the mammary glands of dairy cows; whereas, *cis*-9 C16:1 in milk comes from endogenous synthesis using C16:0 regulated by stearoyl CoA desaturase [149]. In addition, *trans* C16:1 in milk fat is a product of the isomerization of dietary *trans-3* C16:1, *cis*-9 C16:1, or the oxidation of C18:1 ruminal bio-hydrogenation intermediates [150]. Increase in *trans* FA in milk of dairy cows fed camelina oil occurs mainly due to *trans*-11 C18:1, a common intermediate product of ruminal bio-hydrogenation of PUFA [151]. Increased *trans* FA in milk of dairy cows fed camelina oil is attributable to incomplete bio-hydrogenation of unsaturated FA in the rumen [148]. In a nutshell, researchers have reported an increase in desirable FA (e.g., MUFA, PUFA, CLA, and n-3 and n-6 PUFA) and a decrease in unhealthy SFA in milk obtained from cows fed diets with camelina seeds or its derivatives [2,7,10]. However, increase in the trans FA isomers which are unhealthy should be considered while increasing the inclusion rate of camelina seed or its by-products [7].

## 8. Conclusions

To summarize, *C. sativa* is an oilseed plant, the seeds and derivatives of which can be used in ruminant diets with minimal negative consequences, as replacement of conventionally used protein sources. The nutritional profile of *C. sativa* can be enhanced, in terms of a better fatty and amino acid profile and reduced anti-nutritional factors, with the help of mechanical, chemical, and genetic engineering techniques. The use of camelina seed and its by-products in dairy cow diets reduces ruminal cellulolytic bacteria and biohydrogenation, resulting in an increase in beneficial FA (MUFA, PUFA, CLA, n-3, n-6 FA) and decreased SFA levels in the milk of dairy cows. At optimum inclusion levels, *C. sativa* and its derivatives can be utilized safely in dairy cow feed. However, special attention should be given to suppression in feed intake and lowered acetate production that may decrease milk fat percentage and give rise to greater levels of undesirable trans FA isomers in milk fat. Further studies are required to corroborate the optimal inclusion level of *C. sativa* and its derivatives in rations of dairy cows for minimal negative consequences. To establish acceptable dietary inclusion levels, further in vivo experiments are required to evaluate *C. sativa* for a wider range of animals at different physiological stages.

## Figures and Tables

**Table 1:** Chemical composition of *Camelina sativa* seed, meal, expeller, and cake (on DM basis).

Item	Camelina Seed	Camelina Meal	Camelina Expeller	Camelina Cake
Moisture (%)	6.59–11.4	6–8.85	6.55–11.8	7.2–9.11
Dry matter (%)	88.6–93.41	91.15–94	88.2–93.45	90.89–92.8
Crude Protein (%)	27–34	26.5–41.1	19.35–35.70	38.42
NE_L_ Mcal/kg of DM	2.58	2.02	-	-
Digestible Energy (kcal/kg)	-	-	2172	-
Gross Energy (kcal/kg)	5139	5429	-	50.57–51.97
Organic matter (%)	-	-	-	-
Crude Fiber	-	-	-	12–16.92
Ether Extract	18	-	-	10.52–12.70
ADF	14.68–15.1	11.1–19.3	18.8	17.2–22.53
NDF	28.6–30.24	23.3–39.9	22.7–29.2	25.4–38.3
References	[7,15,22,25]	[7,8,9,14,26]	[2,18,20,22,24]	[16,17,21]

**Table 2:** Mineral profile of *Camelina sativa* meal, seed, cake, and cold pressed meal (on DM basis).

Mineral	Camelina Meal	Camelina Seed	Camelina Cake	Cold Pressed Camelina Meal
Macro Minerals (ppm)				
P	7468–10,000	7450–7800	6800–8000	9700–11,000
K	10,788–15,000	8600	-	13,200
Ca	2100–3300	2600–3700	2800–3100	3300–3600
Mg	3794–5000	3400–4053	-	5000
S	6723–11,200	6100	-	9900
Na	14.5–100	-	-	<0.015
**Micro Minerals (ppm)**				
Fe	148–267	95.51–145	-	133.42
Mn	13.94–25.2	23.15	-	34.73
Zn	50.42–50.90	42.15–70.8	-	67.7
Cu	6.55–7.52	6.74–12	-	9.79
Al	1.78–35.16	-	-	-
Cl	2000	-	-	-
References	[5,8,14,28]	[23,25]	[19]	[21,23]

**Table 3:** Fatty acid profile of *Camelina sativa* seed, meal, expeller, cake, and forage (% total fatty acids).

Fatty Acid, %	Camelina Seed	Camelina Meal	Camelina Expeller	Camelina Cake	Camelina Forage	Camelina Oil
Myristic (C14:0)	0.09–0.2	0.17	-	0.13–0.14	0.63	0.06
Pentadecylic (C15:0)	-	-	-	005–0.06	-	-
Palmitic acid, (C16:0)	5.1–10.3	9.12–9.19	7.22–14	7.19–7.46	18.59	5.2–7.00
Palmitoleic (*cis-*9 C16:1)	0.1–0.9	0.32-.52	0.16	-	-	0.08
Stearic (C18:0)	2.19–2.81	2.27–2.9	2.02–2.64	-	0.1	2.2–3.08
Elaidic (*trans*-9 C18:1)	12.14–19	-	13.14	-	-	10.57–19.37
Oleic (C18:1)	11.9–19.9	17.71–21.7	23.7	-	7.9	15.10–18.70
Linoleic (C18:2)	13.5–20.9	24.35–28.8	22.34–31.1	-	13.5	16.00–19.60
Linolenic (C18:3)	28.6–41.3	24.2–46.3	14.3–31.98	-	43.25	28.00–38.10
Arachidic (C20:0)	1.2–1.8	1.17	0.81–15.3	-	3.23	1.22–2.33
Eicosenoic (C20:1)	13.3–25.4	10.1–13.3	11.93	-	-	11.60–15.1
Gondoic (C20:1 n-9)	11.9–15.57	11.23–13.3	-	10.18–10.56	0.2	10.56–15.19
Behenoic (22:0)	0.3–6.2	-	3.4	0.36–0.38	0.75	0.26–0.44
Erucic (C22:1 n-9)	1.6–4.2	0.77	2.86	2.84–3.32	-	1.6–4.2
Lignoceric (C24:0)	0.2	-	-	0.25–0.28	-	0.13–0.28
Nervonic (C24:1 n-9)	0.6–0.7	-	-	0.64–0.8	-	0.48–0.79
Total SFA	9.04–13.13	9.67–9.86	-	-	-	10.2–11.3
Total MUFA	31.0–37.7	33.5–33.87	-	-	-	31.6–34.6
PUFA	51.8–57.4	-	-	-	-	55.2
References	[7,11,30,31,32,33,35,38,39,48,49,50,51]	[7,14,31,34,36]	[2,11]	[19]	[13]	[40,50,52,53,54,55,56,57,58,59]

**Table 4:** Amino acid profile of *Camelina sativa* seed, meal, expeller, and cake (% total amino acids).

Amino Acid, %	Camelina Seed	Camelina Meal	Camelina Expeller	Camelina Cake
Arginine	8.15–8.57	2.81–4.06	2.75–2.99	2.90–3.45
Histidine	2.60–4.06	0.6–2.02	0.78–1.01	0.85–1.09
Isoleucine	3.96–4.62	1.1–2.13	1.18–1.21	1.29–1.62
Leucine	6.63–7.12	1.77–3.32	2.14–2.24	2.34–2.70
Lysine	4.52–4.46	1.35–2.4	1.57–1.67	1.77–2.07
Methionine	1.72–2.85	0.6–1.26	0.61–0.63	0.63–0.73
Phenylalanine	4.19–5.22	1.1–2.37	1.4–1.48	0.48–1.74
Threonine	2.75–2.89	1.08–1.86	1.32–1.38	1.46–1.64
Valine	5.42–6.34	1.54–3.14	1.72–1.86	1.80–2.17
Alanine	4.61–6.14	1.28–3.07	1.4–1.45	1.56–1.87
Aspartic acid	8.71–9.04	2.25–4.36	2.47–2.99	3.22–3.35
Cystine	1.94–2.12	0.64–1.04	0.28–0.85	0.30–0.90
Glutamic acid	14.98–16.12	4.26–7.43	5.34–6.07	6.54–6.81
Glycine	5.25–6.06	1.36–3.44	1.7–1.84	1.98–2014
Proline	5.09–6.07	1.93–3.02	1.63–1.98	2.11–2.13
Serine	5.04–5.96	1.15–3.23	1.21–1.48	1.3–1.74
Tyrosine	3.04–3.64	0.63–1.82	0.9–0.97	0.97–1.14
References	[60,61]	[12,14,21,23]	[18,22]	[16,17,19]

**Table 5:** Comparison of camelina meal with other oilseed meals.

Item (%)	Camelina Meal	Canola Meal	Soybean Meal	Rapeseed Meal
Dry matter	92.2	91.4	90.2	87.7
Organic matter	93.9	92.2	92.7	-
Crude protein	41.9	39.4–40.1	49.6–54.9	34.5
NDF	33.4	28.5	10.3–18.8	31.8
ADF	23.8	19.4–27.6	6.2–19.5	21.6
Ether extract	7.03	4.56	1.1–1.92	4.11
Ash	5.98	7.69	6.9	8.4
Amino acids, % CP				
Histidine	1.72	2.52	2.55	2.13
Isoleucine	2.17	3.53	3.89	3.76
Leucine	3.24	6.39	7.52	6.49
Lysine	2.27	4.87	5.91	4.8
Methionine	1.08	1.88	1.55	1.56
Phenylalanine	2.27	3.74	5.02	3.59
Threonine	1.59	3.87	4.07	4.32
Tryptophan	-	1.35	-	1.14
Valine	2.81	4.47	3.76	4.77
Arginine	4.13	5.9	-	5.58
Alanine	2.81	4.43	4.26	4.16
Glycine	3	5.13	4.21	4.54
Proline	2.98	6.2	5.07	5.45
Serine	2.81	4.13	5.44	4.11
Tyrosine	0.78	2.9	3.66	2.85
Glutamic acid	7.6	22.7	14.99	15.6
Cysteine	0.94	2.43	1.47	2.58
Aspartic acid	4.35	7.34	11.43	6.78
Minerals, % DM				
Ca	0.31	0.89	0.7	0.8
P	0.82	1.11	0.73	1.1
References	[5]	[5,9,101]	[101,102,103,104]	[105,106]

**Table 6:** Anti-nutritional factors of *Camelina sativa* cake, expeller, and meal.

Antinutritional Factors	Meals of Different Genotypes of *Camelina sativa*						Screw-Pressed Camelina Cake	Camelina Expellers	Camelina Meal	
	CS Lindo	CS Ukrajin Skaja	CS Soledo	CS Volynskaja	CS Zarja Socialisma	CS Bavaria				
GSL (mmol kg^−1^)	21.9	23.7	23.1	24.3	23.6	19.9	36.3	36.3	-	-
Phytic acid (g kg^−1^)	24.1	22.2	21	24.8	12	22.2	-	-	-	40.7
Condensed tannins (g kg^−1^)	2.11	1.98	1.81	2.09	2.11	1.89	2	1.9	-	34.2
Sinapine (g kg^−1^)	2.19	3.04	2.64	3.27	2.55	2.56	-	-	-	-
GSL-9 (mg g^−1^)	-	-	-	-	-	-	-	-	3.48	-
GSL-10 (mg g^−1^)	-	-	-	-	-	-	-	-	7.72	-
GSL-11 (mg g^−1^)	-	-	-	-	-	-	-	-	1.25	-
Total GSL (mg g^−1^)	-	-	-	-	-	-	-	-	12.45	-
References	[12]						[16]	[18]	[8]	[108]

CS = *Camelina sativa*; GSL = Glucosinolates.

**Table 7:** Effect of the Camelina seed and its byproducts on feed intake, rumen digestibility and VFAs production in dairy cattle.

Treatment	Inclusion Rate ^1^	Study Type	DMI, Kg/d	OM Digestibility, %	DM Digestibility, %	NDF, %	ADF, %	Protein, %	pH	TVFA, mM	Acetate, mmol/100 mmol	Butyrate, mmol/100 mmol	Propionate, mmol/100 mmol	Acetate: Propionate	References
CS	2.9 ^x^	In vivo	20.6	-	-	-	-	-	6.02	-	54.9 ^b^*	16.6	21.9 ^b^*	-	[7]
CM	9.5		19.8	-	-	-	-	-	6.02	-	51.4 ^c^*	16.1	25.5 ^a^*	-	
Control	0.00		21.0	-	-	-	-	-	6.11	-	57.7 ^a^**	15.6	21.2 ^c^*	-	
CO	2.9 ^y^	In vivo	23.3 *	68.5	-	54.6	-	-	-	-	-	-	-	-	[2]
CE	20		22.7 *	68.0	-	52.5	-	-	-	-	-	-	-	-	
Control	0		23.3	69.9	-	55.0	-	-	-	-	-	-	-	-	
CM	10	In vivo	4.91	66.4 ^a^*	66.5	57.0	53.0	63.3	6.8	80.9	67.6	9.3 ^c^*	20.9	3.25	[8]
LINM	10		4.93	63.6 ^b^*	64.0	56.2	56.6	59.4	6.8	78.0	67.1	9.9 ^b^*	20.8	3.24	
DDGS	10		5.10	64.7 ^ab^*	65.0	56.9	56.3	60.8	6.7	77.5	66.6	11.0 ^a^*	20.8	3.32	
CM0	0	In vitro	-	51.1	45.0	52.5 *	32.2	54.8	-	76.5	63.7 ***	14.1	19.4 ***	3.28 ***	[9]
CM50	10.1		-	48.4	44.5	48.0 *	32.6	51.1	-	78.9	57.2 ***	13.6	25.9 ***	2.22 ***	
CM100	20.2		-	47.2	43.2	45.0 *	29.7	53.4	-	77.6	54.9 **	14.2	27.9 **	1.98 **	
CS	4.2	In vivo	24.2	-	-	-	-	-	6.31	-	60.52	10.32	25.58	2.40	[11]
CE	9.5		24.4	-	-	-	-	-	6.25	-	60.52	11.10	26.00	2.38	
DDGS	18		23.7	-	-	-	-	-	6.37	-	62.31	12.55	23.42	2.86	
WFS	4.7		25.9	-	-	-	-	-	6.28		61.53	10.56	23.79	2.55	
CM		In vitro	-	44.55	-	-	-	-	6.79	62.11 *	35.79 *	7.51	12.47	2.88	[3]
SBM			-	47.04	-	-	-	-	6.92	72.58 *	42.91 *	8.17	14.10	3.05	
CS (5% EE)	7.7	In vitro	-	53.9 *	50.2 *	47.0 *	40.1 *	49.2 *	-	85.1 **	49.1 ^c^**	14.9	30.1 **	1.64 ^c^*	[115]
CS (8%EE)	17.7		-	53.3	48.1	44.8	36.7	51.5	-	79.4	50.1 ^c^**	14.9	28.9	1.77 ^c^*	
CaPO	5% EE		-	58.7 *	56.1 *	56.1 *	49.1 *	55.5 *	-	89.9 **	55.8 ^b^**	14.6	25.9 **	2.21 ^b^*	
	8% E.E.		-	57.1	54.9	57.6	52.4	56.9	-	89.1	60.6 ^a^**	12.9	23.5	2.60 ^a^*	

DMI = Dry Matter Intake; OM = Organic Matter; DM = Dry Matter; NDF = Neutral Degradable Fiber; ADF = Acid degradable Fiber; TVFA = Total Volatile Fatty Acids, CS = Camelina Seed; CM = Camelina Meal; CO = Camelina Oil; CE = Camelina Expeller; DDGS = Distiller Dried Grains with Solubles; LINM = Linseed Meal; WFS = Whole Flax Seed; SBM = Soybean Meal; EE = Ether Extract; CaPO = Calcium salt of Palm Oil, 1 = % DM basis unless otherwise indicated; x = % in diet (DM basis); y = % in concentrate (DM basis), ^a–c^, *, **, *** = Values with superscripts describe the significant difference (* = *p* < 0.05, ** = *p* < 0.01, *** = *p* < 0.001).

**Table 8:** Effect of the Camelina seed and its by-products on milk production and composition in dairy cow’s milk.

Treatment	Inclusion Rate (% DM Basis)	Milk Production (kg/d)	Lactose (g/d)	Protein (g/d)	Fat (g/d)	Lactose (%)	Protein (%)	Fat (%)	References
CS	2.9% ^x^	34.40	-	967	865 ^b^***	4.86	2.83	2.51 ^b^***	[7]
CM	9.5%	32.50	-	902	481 ^c^***	4.75	2.76	1.44 ^c^***	
Control	0.00	33.80	-	980	1063 ^a^***	4.93	2.89	3.14 ^a^***	
CO	2.9% ^y^	31.20	1450	992	1234	4.61	3.23	3.93	[2]
CE	20%	32.20	1485	1014	1192	4.61	3.15	3.67	
Control (0)	0	31.10	1431	1013	1225	4.60	3.30	3.96	
CS	4.2%	36.50 *	1699	1161	1258 ^b^**	4.66	3.20	3.48 ^b^**	[11]
CE	9.5%	37.00	1729	1133	1000 ^c^**	4.69	3.07	2.71 ^c^**	
DDGS	18%	37.40	1748	1182	1355 ^a^**	4.66	3.16	3.63 ^a^**	
WFS	4.7%	35.60 *	1652	1146	1328	4.64	3.22	3.74	
CM	0% (31% SFM)	19.27	-	-	-	4.45	2.85	3.39	[10]
CM50	50% (15.5 SFM + 15.5% CM)	18.35	-	-	-	4.45	2.91	3.17	
CM100	100 (30.1% CM)	19.63	-	-	-	4.45	2.95	3.16	

CS = Camelina Seed; CM = Camelina Meal; CO = Camelina Oil; CE = Camelina Expeller; DDGS = Distiller Dried Grains with Solubles; WFS = Whole Flax Seed; x = % in diet (DM basis); y = % in concentrate (DM basis), ^a–c^, *, **, *** = Values with superscripts describe the significant difference (* = *p* < 0.05, ** = *p* < 0.01, *** = *p* < 0.001).

**Table 9:** Effect of Camelina seed and derivatives on the composition of milk fatty acid composition in dairy Cow’s milk fat.

Treatment	Inclusion Rate (%, DM Basis)	SFA (%)	MUFA (%)	PUFA (%)	n-3 FA (%)	n-6 FA (%)	n-6/n-3	References
CS	2.9% ^x^	66.6 ^b^***	30.7 ^b^***	2.70 ^a^*	-	-	-	[7]
CM	9.5%	57.4 ^c^***	39.7 ^a^***	2.92 ^a^*	-	-	-	
Control	0.00	72.7 ^a^***	25.1 ^c^***	2.16 ^b^*	-	-	-	
CO	2.9% ^y^	65.4 ^b^***	28.2 ^b^***	5.93 ^b^***	-	-	-	[2]
CE	20%	62.6 ^c^***	29.7 ^a^*	7.27 ^a^***	-	-	-	
Control	0	71.0 ^a^***	23.2 ^c^***	5.33 ^c^***	-	-	-	
CM	0% (31% SFM)	64.98 ***	27.67 **	5.52 ***	0.62	4.92 ***	9.45 *	[10]
CM50	50% (15.5 SFM + 15.5% CM)	61.87 ***	29.58 **	6.43 ***	0.61	5.81 ***	9.60 *	
CM100	100 (30.1% CM)	60.34 ***	30.48 **	6.31 ***	0.67	6.64 ***	10.25 *	

FA = Fatty Acids; SFA = Total Saturated FA; MUFA = Mono-Unsaturated FA; PUFA = Poly-Unsaturated FA; n-3 = Total n-3 FA, n-6 = Total n-6 FA; CS = Camelina Seed; CM = Camelina Meal; CO = Camelina Oil; CE = Camelina Expeller; x = % in diet (DM basis); y = % in concentrate (DM basis); ^a–c^, *, **, *** = Values with superscripts describe the significant difference (* = *p* < 0.05, ** = *p* < 0.01, *** = *p* < 0.001).

**Table 10:** Effect of camelina seed and its derivatives on the fatty acid composition of milk fat in dairy cows.

FA (% of Total FA)	Hurtaud and Peyraud [7] ^x^			Toma et al. [10]			Halmemies-Beauchet-Filleau et al. [2] ^y^			Bayat et al. [40] ^1^	
	Control	CS (2.9%)	CM (9.5%)	Control	CM50	CM100 ^1^	Control	CO (2.9%)	CE (20%)	Control	CO (6%)
C4:0	2.44	2.16	1.40	0.11	0.08	0.06	3.35	3.57	3.67	3.10	3.18
C5:0	0.023	0.025	0.024	-	-	-	-	-	-	-	-
C6:0	1.95	1.84	0.99	-	-	-	1.76	1.69	1.69	1.90	1.56
C7:0	0.020	0.022	0.019	-	-	-	-	-	-	-	-
C8:0	1.34	1.27	0.61	-	-	-	1.23	1.14	1.09	1.12	0.79
C9:0	0.048	0.037	0.029	-	-	-	-	-	-	-	-
C10:0	3.33	3.16	1.59	-	-	-	3.19	2.72	2.57	2.66	1.55
*cis*-9 C10:1	-	-	-	-	-	-	0.30	0.27	0.26	0.296	0.193
C11:0	0.085	0.058	0.054	-	-	-	-	-	-	-	-
C12:0	4.13	4.04	2.61	3.46	3.46	3.46	3.91	3.20	3.09	3.24	1.84
*cis*-9 C12:1	-	-	-	-	-	-	0.09	0.07	0.07	0.082	0.043
*trans*-9 C12:1	-	-	-	-	-	-	0.08	0.07	0.07	0.081	0.046
C13:0	0.132	0.130	0.131	-	-	-	-	-	-	-	-
C14:1	1.28	1.42	1.96	12.66	12.67	12.44	-	-	-	-	-
C14:0	12.99	12.95	11.77	-	-	-	13.0	11.6	11.9	12.1	8.10
*cis*-9 C14:1	-	-	-	-	-	-	0.97	0.85	0.99	1.12	0.77
*trans*-9 C14:1	-	-	-	-	-	-	0.014	0.012	0.014	0.230	0.456
*Iso*-C15	0.24	0.23	0.23	-	-	-	-	-	-	-	-
C15:1	0.57	0.53	0.54	-	-	-	-	-	-	-	-
C15:0	1.35	1.21	1.42	-	-	-	2.22	1.92	1.98	2.36	1.55
C16:0	2.14	2.25	4.14	31.01	20.03	28.20	32.4	27.1	26.8	34.4	21.3
C16:1	37.0	32.2	31.9	1.50	1.39	1.38	1.89	1.67	1.93	2.43	2.10
*cis* C16:1	-	-	-	-	-	-	1.60	1.36	1.52	2.20	1.64
*trans* C16:1	-	-	-	-	-	-	0.29	0.21	0.31	0.230	0.456
*Iso*-C17	0.40	0.41	0.57	-	-	-	-	-	-	-	-
C17:1	0.91	0.88	1.04	-	-	-	-	-	-	-	-
C17:0	0.58	0.54	0.59	-	-	-	1.21	1.11	1.23	1.18	0.86
C18:3	0.20	0.32	0.36	-	-	-	-	-	-	-	-
*trans*-6-8 C18:1	0.26	0.57	0.64	-	-	-	-	-	-	-	-
*cis* C18:1	-	-	-	-	-	-	14.5	18.1	15.7	17.5	25.3
*trans* C18:1	-	-	-	-	-	-	4.02	4.91	8.28	2.31	6.71
*trans*-9, *trans*-12 C18:2	0.09	0.18	0.67	-	-	-	-	-	-	-	-
*cis*-9, *cis*-12 C18:2	1.86	2.20	1.89	-	-	-	-	-	-	-	-
CLA	-	-	-	-	-	-	0.59	0.79	1.33	0.38	0.95
C18:3 n-3	-	-	-	-	-	-	1.10	1.17	1.06	0.454	0.489
C18:3 n-6	-	-	-	-	-	-	0.05	0.06	0.05	0.015	0.007
*cis*-9,*truns*-11, *cis*-15 C18:3	-	-	-	-	-	-	0.03	0.05	0.05	0.036	0.056
*cis*-9,*trans*-11,*trans*-15 C18:3	-	-	-	-	-	-	0.014	0.023	0.055	-	-
C18:4 n-3	-	-	-	-	-	-	0.02	0.02	0.02	-	-
*trans*-9 C18:1	0.26	0.55	0.58	23.05	25.13	25.34	-	-	-	-	-
*trans*-10 C18:1	1.02	3.44	11.27	-	-	-	-	-	-	-	-
*trans*-11 C18:1	1.26	2.19	3.34	-	-	-	-	-	-	-	-
*trans*-12 C18:1	0.15	0.18	0.52	-	-	-	-	-	-	-	-
*cis*-9 C18:1	16.4	17.3	14.1	-	-	-	-	-	-	-	-
*trans*-15, *cis*-11	0.68	1.03	1.47	-	-	-	-	-	-	-	-
C18:1	-	-	-	-	-	-	18.5	23.0	24.0	-	-
C18:1 *cis*-12	0.23	0.43	0.07	-	-	-	-	-	-	-	-
C18:0	6.61	6.09	3.40	9.16	9.17	8.85	-	-	-	8.78	12.9
C20:0	-	-	-	-	-	-	0.42	0.77	0.57	0.178	1.69
*cis* C20:1	-	-	-	-	-	-	0.50	1.24	1.20	0.247	2.48
*trans* C20:1	-	-	-	-	-	-	0.08	0.23	0.29	0.040	0.585
C20:1	-	-	-	-	-	-	0.58	1.47	1.49	0.287	3.07
C20:2 n-6	-	-	-	-	-	-	0.045	0.073	0.088	0.024	0.063
C20:3 n-3	-	-	-	-	-	-	0.020	0.037	0.037	0.008	0.046
C20:3 n-6	-	-	-	-	-	-	0.093	0.087	0.073	0.047	0.033
C20:4 n-3	-	-	-	-	-	-	0.09	0.07	0.08	0.034	0.026
C20:4 n-6	-	-	-	-	-	-	0.07	0.08	0.07	0.066	0.046
C20:5 n-3	-	-	-	-	-	-	0.13	0.11	0.10	0.049	0.032
C22:0	-	-	-	-	-	-	0.10	0.12	0.10	0.054	0.157
C22:1	-	-	-	-	-	-	0.07	0.19	0.18	0.025	0.286
C22:2 n-6	-	-	-	-	-	-	0.006	0.008	0.012	-	-
C22:3 n-3	-	-	-	-	-	-	0.003	0.012	0.015	-	-
C22:4 n-6	-	-	-	-	-	-	0.018	0.016	0.015	0.018	0.013
C22:5 n-3	-	-	-	-	-	-	0.074	0.071	0.060	0.060	0.038
C22:6 n-3	-	-	-	-	-	-	0.004	0.003	0.003	-	-
C26:0	-	-	-	-	-	-	0.013	0.011	0.012	0.030	0.013
C28:0	-	-	-	-	-	-	0.003	0.003	0.004	-	-
*trans* FA	-	-	-	-	-	-	6.56	8.47	11.7	3.37	11.8
SFA	72.7	66.6	57.4	64.98	61.87	60.34	71.0	65.4	62.6	72.1	56.0
UFA	27.3	33.4	42.6	-	-	-	-	-	-	-	-
MU FA	25.1	30.7	39.7	27.67	29.58	30.48	23.2	28.2	29.7	24.7	39.1
PUFA	2.16	2.70	2.92	5.52	6.43	6.31	5.33	5.93	7.27	2.89	4.46

FA = Fatty Acids; SFA = Total Saturated FA; MUFA = Monounsaturated FA; PUFA = Polyunsaturated FA; CLA = Conjugated linoleic Acid; CS = Camelina Seed; CM = Camelina Meal; CO = Camelina Oil; CE = Camelina Expeller; 1 = % DM basis unless otherwise indicated; x = % in diet (DM basis); y = % in concentrate (DM basis).

## Data Availability

Not applicable.

## References

[1] Mottet A., de Haan C., Falcucci A., Tempio G., Opio C., Gerber P. (2017). Livestock: On our plates or eating at our table? A new analysis of the feed/food debate. Glob. Food Sec..

[2] Halmemies-Beauchet-Filleau A., Kokkonen T., Lampi A.-M., Toivonen V., Shingfield K., Vanhatalo A. (2011). Effect of plant oils and camelina expeller on milk fatty acid composition in lactating cows fed diets based on red clover silage. J. Dairy Sci..

[3] Sizmaz Ö., Çalik A., Bundur A. (2021). In Vitro Fermentation Characteristics of Camelina Meal Comparison with Soybean Meal. Livest. Stud..

[4] Waraich E.A., Ahmed Z., Ahmad R., Ashraf M.Y., Naeem M.S., Rengel Z. (2013). ‘*Camelina sativa*’, a climate proof crop, has high nutritive value and multiple-uses: A review. Aust. J. Crop Sci..

[5] Paula E.M., da Silva L.G., Brandao V.L.N., Dai X., Faciola A.P. (2019). Feeding canola, camelina, and carinata meals to ruminants. Animals.

[6] Czerniawski P., Piasecka A., Bednarek P. (2021). Evolutionary changes in the glucosinolate biosynthetic capacity in species representing Capsella, Camelina and Neslia genera. Phytochemistry.

[7] Hurtaud C., Peyraud J.-L. (2007). Effects of feeding camelina (seeds or meal) on milk fatty acid composition and butter spreadability. J. Dairy Sci..

[8] Lawrence R., Anderson J., Clapper J. (2016). Evaluation of camelina meal as a feedstuff for growing dairy heifers. J. Dairy Sci..

[9] Brandao V., Silva L., Paula E., Monteiro H., Dai X., Lelis A., Faccenda A., Poulson S., Faciola A. (2018). Effects of replacing canola meal with solvent-extracted camelina meal on microbial fermentation in a dual-flow continuous culture system. J. Dairy Sci..

[10] Toma S., Dragomir C., Habeanu M., Ropota M., Cismileanu A., Grosu H. (2015). Effects of replacing sunflower meal with camelina meal on dairy cows performances. Arch. Zootech..

[11] Sarramone J., Gervais R., Benchaar C., Chouinard P. (2020). Lactation performance and milk fatty acid composition of lactating dairy cows fed *Camelina sativa* seeds or expeller. Anim. Feed Sci. Technol..

[12] Colombini S., Broderick G.A., Galasso I., Martinelli T., Rapetti L., Russo R., Reggiani R. (2014). Evaluation of *Camelina sativa* (L.) Crantz meal as an alternative protein source in ruminant rations. J. Sci. Food Agric..

[13] Colonna M.A., Giannico F., Tufarelli V., Laudadio V., Selvaggi M., De Mastro G., Tedone L. (2021). Dietary Supplementation with *Camelina sativa* (L. Crantz) Forage in Autochthonous Ionica Goats: Effects on Milk and Caciotta Cheese Chemical, Fatty Acid Composition and Sensory Properties. Animals.

[14] Aziza A., Panda A., Quezada N., Cherian G. (2013). Nutrient digestibility, egg quality, and fatty acid composition of brown laying hens fed camelina or flaxseed meal. J. Appl. Poult. Res..

[15] Peng Q., Khan N.A., Wang Z., Yu P. (2014). Moist and dry heating-induced changes in protein molecular structure, protein subfractions, and nutrient profiles in camelina seeds. J. Dairy Sci..

[16] Kahindi R.K., Woyengo T.A., Thacker P., Nyachoti C. (2014). Energy and amino acid digestibility of camelina cake fed to growing pigs. Anim. Feed Sci. Technol..

[17] Woyengo T., Patterson R., Levesque C. (2018). Nutritive value of multienzyme supplemented cold-pressed camelina cake for pigs. J. Anim. Sci..

[18] Kiarie E., Walsh M., He L., Velayudhan D., Yin Y., Nyachoti C. (2016). Phytase improved digestible protein, phosphorous, and energy contents in camelina expellers fed to growing pigs. J. Anim. Sci..

[19] Smit M., Beltranena E. (2017). Effects of feeding camelina cake to weaned pigs on safety, growth performance, and fatty acid composition of pork. J. Anim. Sci..

[20] Ye C.L., Anderson D.M., Lall S.P. (2016). The effects of camelina oil and solvent extracted camelina meal on the growth, carcass composition and hindgut histology of *Atlantic salmon* (*Salmo salar*) parr in freshwater. Aquaculture.

[21] Woyengo T., Patterson R., Slominski B., Beltranena E., Zijlstra R. (2016). Nutritive value of cold-pressed camelina cake with or without supplementation of multi-enzyme in broiler chickens. Poult. Sci..

[22] Ryhänen E.L., Perttilä S., Tupasela T., Valaja J., Eriksson C., Larkka K. (2007). Effect of *Camelina sativa* expeller cake on performance and meat quality of broilers. J. Sci. Food Agric..

[23] Kasiga T., Karki B., Croat J., Kaur J., Gibbons W.R., Muthukumarappan K., Brown M.L. (2020). Process effects on carinata Brassica carinata and camelina *Camelina sativa* seed meal compositions and diet palatability in Rainbow Trout Oncorhynchus mykiss. Anim. Feed Sci. Technol..

[24] Halmemies-Beauchet-Filleau A., Rinne M., Lamminen M., Mapato C., Ampapon T., Wanapat M., Vanhatalo A. (2018). Alternative and novel feeds for ruminants: Nutritive value, product quality and environmental aspects. Animal.

[25] Zając M., Kiczorowska B., Samolińska W., Klebaniuk R. (2020). Inclusion of camelina, flax, and sunflower seeds in the diets for broiler chickens: Apparent digestibility of nutrients, growth performance, health status, and carcass and meat quality traits. Animals.

[26] Moriel P., Nayigihugu V., Cappellozza B., Gonçalves E., Krall J., Foulke T., Cammack K., Hess B. (2011). Camelina meal and crude glycerin as feed supplements for developing replacement beef heifers. J. Anim. Sci..

[27] Zubr J. (2010). Carbohydrates, vitamins and minerals of *Camelina sativa* seed. Nutr. Food Sci..

[28] Cherian G., Campbell A., Parker T. (2009). Egg quality and lipid composition of eggs from hens fed Camelina sativa. J. Appl. Poult. Res..

[29] Zubr J. (2009). Unique dietary oil from *Camelina sativa* seed. Agrafood Ind. Hi-Tech.

[30] Yang J., Caldwell C., Corscadden K., He Q.S., Li J. (2016). An evaluation of biodiesel production from *Camelina sativa* grown in Nova Scotia. Ind. Crops Prod..

[31] Quezada N., Cherian G. (2012). Lipid characterization and antioxidant status of the seeds and meals of *Camelina sativa* and flax. Eur. J. Lipid Sci. Technol..

[32] Wu X., Leung D.Y. (2011). Optimization of biodiesel production from camelina oil using orthogonal experiment. Appl. Energy.

[33] Budin J.T., Breene W.M., Putnam D.H. (1995). Some compositional properties of camelina (*Camelina sativa* L. Crantz) seeds and oils. J. Am. Oil Chem. Soc..

[34] Petre S.M., Moraru A., Dobre P., Jurcoane S. (2015). Life Cycle Assessment of *Camelina sativa*—Environmental friendly source for biofuels and livestock protein available in Romania. Rom. Biotechnol. Lett..

[35] Zubr J., Matthäus B. (2002). Effects of growth conditions on fatty acids and tocopherols in *Camelina sativa* oil. Ind. Crops Prod..

[36] Cappellozza B.I., Cooke R., Bohnert D., Cherian G., Carroll J. (2012). Effects of camelina meal supplementation on ruminal forage degradability, performance, and physiological responses of beef cattle. J. Anim. Sci..

[37] Abramovič H., Butinar B., Nikolič V. (2007). Changes occurring in phenolic content, tocopherol composition and oxidative stability of *Camelina sativa* oil during storage. Food Chem..

[38] Krzyżaniak M., Stolarski M.J., Tworkowski J., Puttick D., Eynck C., Załuski D., Kwiatkowski J. (2019). Yield and seed composition of 10 spring camelina genotypes cultivated in the temperate climate of Central Europe. Ind. Crops Prod..

[39] Ebeid H.M., Hassan F.-U., Li M., Peng L., Peng K., Liang X., Yang C. (2020). *Camelina sativa* L. Oil Mitigates Enteric in vitro Methane Production, Modulates Ruminal Fermentation, and Ruminal Bacterial Diversity in Buffaloes. Front. Vet. Sci..

[40] Bayat A., Kairenius P., Stefański T., Leskinen H., Comtet-Marre S., Forano E., Chaucheyras-Durand F., Shingfield K. (2015). Effect of camelina oil or live yeasts (*Saccharomyces cerevisiae*) on ruminal methane production, rumen fermentation, and milk fatty acid composition in lactating cows fed grass silage diets. J. Dairy Sci..

[41] Mupondwa E., Li X., Tabil L., Falk K., Gugel R. (2016). Technoeconomic analysis of camelina oil extraction as feedstock for biojet fuel in the Canadian Prairies. Biomass Bioenergy.

[42] Blackshaw R., Johnson E., Gan Y., May W., McAndrew D., Barthet V., McDonald T., Wispinski D. (2011). Alternative oilseed crops for biodiesel feedstock on the Canadian prairies. Can. J. Plant Sci..

[43] Singh B.K., Bala M., Rai P.K. (2014). Fatty acid composition and seed meal characteristics of Brassica and allied genera. Natl. Acad. Sci. Lett..

[44] Zubr J. (1997). Oil-seed crop: *Camelina sativa*. Ind. Crops Prod..

[45] Kurasiak-Popowska D., Ryńska B., Stuper-Szablewska K. (2019). Analysis of distribution of selected bioactive compounds in *Camelina sativa* from seeds to pomace and oil. Agronomy.

[46] Peiretti P., Meineri G. (2007). Fatty acids, chemical composition and organic matter digestibility of seeds and vegetative parts of false flax (*Camelina sativa* L.) after different lengths of growth. Anim. Feed Sci. Technol..

[47] Pavlista A., Hergert G., Margheim J., Isbell T. (2016). Growth of spring camelina (*Camelina sativa*) under deficit irrigation in Western Nebraska. Ind. Crops Prod..

[48] Raczyk M., Popis E., Kruszewski B., Ratusz K., Rudzińska M. (2016). Physicochemical quality and oxidative stability of linseed (*Linum usitatissimum*) and camelina (*Camelina sativa*) cold-pressed oils from retail outlets. Eur. J. Lipid Sci. Technol..

[49] Bonjean A., Goffic F.L. (1999). The sector today and tomorrow-Plant biology-Camelina-*Camelino sativo* (L.) Crantz: An opportunity for European agriculture and industry. OCL-Oleagineux-Corps Gras-Lipides.

[50] Abramovič H., Abram V. (2005). Physico-chemical properties, composition and oxidative stability of *Camelina sativa* oil. Food Technol. Biotechnol..

[51] Berti M., Gesch R., Eynck C., Anderson J., Cermak S. (2016). Camelina uses, genetics, genomics, production, and management. Ind. Crops Prod..

[52] Hixson S.M., Parrish C.C., Anderson D.M. (2014). Changes in tissue lipid and fatty acid composition of farmed rainbow trout in response to dietary camelina oil as a replacement of fish oil. Lipids.

[53] Toncea I., Necseriu D., Prisecaru T., Balint L.-N., Ghilvacs M., Popa M. (2013). The seed’s and oil composition of Camelia–first Romanian cultivar of camelina (*Camelina sativa*, L. Crantz). Rom. Biotechnol. Lett..

[54] Moser B.R., Vaughn S.F. (2010). Evaluation of alkyl esters from *Camelina sativa* oil as biodiesel and as blend components in ultra low-sulfur diesel fuel. Bioresour. Technol..

[55] Jurcoane S.D.P., Florea C., Petre S.M., Ropota M. *Camelina sativa*—A useful plant source for renewable jet fuels, human nutrition and animal feed. Proceedings of the Simpozion National Editia Xia Plante medicinale—Prezent si Perspective.

[56] Katar D. (2013). Determination of fatty acid composition on different false flax (*Camelina sativa* (L.) Crantz) Genotypes under Ankara ecological conditions. Turkish J. Field Crop..

[57] Belayneh H.D., Wehling R.L., Cahoon E., Ciftci O.N. (2015). Extraction of omega-3-rich oil from *Camelina sativa* seed using supercritical carbon dioxide. J. Supercrit. Fluids.

[58] Shukla V., Dutta P., Artz W. (2002). Camelina oil and its unusual cholesterol content. J. Am. Oil Chem. Soc..

[59] Hrastar R., Petrisic M.G., Ogrinc N., Kosir I.J. (2009). Fatty acid and stable carbon isotope characterization of *Camelina sativa* oil: Implications for authentication. J. Agric. Food Chem..

[60] Bătrîna Ș.L., Jurcoane Ș., Popescu I., Marin F., Imbrea I.M., Crista F., Pop G., Imbrea F. (2020). *Camelina sativa*: A study on amino acid content. Biotechnol. Lett..

[61] Zubr J. (2003). Qualitative variation of *Camelina sativa* seed from different locations. Ind. Crops Prod..

[62] Almeida F., Htoo J., Thomson J., Stein H.-H. (2013). Amino acid digestibility in camelina products fed to growing pigs. Can. J. Anim. Sci..

[63] Masella P., Martinelli T., Galasso I. (2014). Agronomic evaluation and phenotypic plasticity of *Camelina sativa* growing in Lombardia, Italy. Crop Pasture Sci..

[64] Vollmann J., Moritz T., Kargl C., Baumgartner S., Wagentristl H. (2007). Agronomic evaluation of camelina genotypes selected for seed quality characteristics. Ind. Crops Prod..

[65] Vollmann J., Damboeck A., Eckl A., Schrems H., Ruckenbauer P. (1996). Improvement of *Camelina sativa*, an underexploited oilseed. Progress in New Crops.

[66] Gehringer A., Friedt W., Lühs W., Snowdon R. (2006). Genetic mapping of agronomic traits in false flax (*Camelina sativa* subsp. sativa). Genome.

[67] Ghamkhar K., Croser J., Aryamanesh N., Campbell M., Kon’kova N., Francis C. (2010). Camelina (*Camelina sativa* (L.) Crantz) as an alternative oilseed: Molecular and ecogeographic analyses. Genome.

[68] Pollard M., Martin T.M., Shachar-Hill Y. (2015). Lipid analysis of developing *Camelina sativa* seeds and cultured embryos. Phytochemistry.

[69] Rodríguez-Rodríguez M.F., Sánchez-García A., Salas J.J., Garcés R., Martínez-Force E. (2013). Characterization of the morphological changes and fatty acid profile of developing *Camelina sativa* seeds. Ind. Crops Prod..

[70] Guy S.O., Wysocki D.J., Schillinger W.F., Chastain T.G., Karow R.S., Garland-Campbell K., Burke I.C. (2014). Camelina: Adaptation and performance of genotypes. Field Crops Res..

[71] Gesch R., Archer D., Berti M. (2014). Dual cropping winter camelina with soybean in the northern corn belt. Agron. J..

[72] Sintim H.Y., Zheljazkov V.D., Obour A.K., y Garcia A.G., Foulke T.K. (2015). Influence of nitrogen and sulfur application on camelina performance under dryland conditions. Ind. Crops Prod..

[73] Jiang Y., Caldwell C.D., Falk K.C. (2014). Camelina seed quality in response to applied nitrogen, genotype and environment. Can. J. Plant Sci..

[74] Sintim H.Y., Zheljazkov V.D., Obour A.K., Garcia y Garcia A., Foulke T.K. (2016). Evaluating agronomic responses of camelina to seeding date under rain-fed conditions. Agron. J..

[75] Karčauskienė D., Sendžikienė E., Makarevičienė V., Zaleckas E., Repšienė R., Ambrazaitienė D. (2014). False flax (*Camelina sativa* L.) as an alternative source for biodiesel production. Žemdirbystė.

[76] Razmaitė V., Pileckas V., Bliznikas S., Šiukščius A. (2021). Fatty acid composition of Cannabis sativa, Linum usitatissimum and *Camelina sativa* seeds harvested in lithuania for food use. Foods.

[77] Lafferty R., Rife C., Foster G. (2009). Spring camelina production guide for the Central High Plains.

[78] Wittenberg A., Anderson J.V., Berti M.T. (2019). Winter and summer annual biotypes of camelina have different morphology and seed characteristics. Ind. Crops Prod..

[79] Schuster A., Friedt W. (1998). Glucosinolate content and composition as parameters of quality of Camelina seed. Ind. Crops Prod..

[80] Russo R., Reggiani R. (2012). Antinutritive compounds in twelve *Camelina sativa* genotypes. Am. J. Plant Sci..

[81] Turina E., Prakhova T.Y., Prakhov V. (2019). Assessment of productivity and adaptability of *Camelina Sativa* varieties. IOP Conference Series: Earth and Environmental Science.

[82] Kirkhus B., Lundon A.R., Haugen J.-E., Vogt G., Borge G.I.A., Henriksen B.I. (2013). Effects of environmental factors on edible oil quality of organically grown *Camelina sativa*. J. Agric. Food Chem..

[83] Raziei Z., Kahrizi D., Rostami-Ahmadvandi H. (2018). Effects of climate on fatty acid profile in *Camelina sativa*. Cell. Mol. Biol..

[84] Obour A.K., Obeng E., Mohammed Y.A., Ciampitti I.A., Durett T.P., Aznar-Moreno J.A., Chen C. (2017). Camelina seed yield and fatty acids as influenced by genotype and environment. Agron. J..

[85] Johnson J.M., Gesch R.W. (2013). Calendula and camelina response to nitrogen fertility. Ind. Crops Prod..

[86] Lošák T., Hlusek J., Martinec J., Vollmann J., Peterka J., Filipcik R., Varga L., Ducsay L., Martensson A. (2011). Effect of combined nitrogen and sulphur fertilization on yield and qualitative parameters of *Camelina sativa* [L.] Crtz.(false flax). Acta Agric. Scand. Sect. B-Soil Plant Sci..

[87] Ahmad Z., Anjum S., Skalicky M., Waraich E.A., Muhammad Sabir Tariq R., Ayub M.A., Hossain A., Hassan M.M., Brestic M., Sohidul Islam M. (2021). Selenium alleviates the adverse effect of drought in oilseed crops Camelina (*Camelina sativa* L.) and Canola (*Brassica napus* L.). Molecules.

[88] Wittkop B., Snowdon R., Friedt W. (2009). Status and perspectives of breeding for enhanced yield and quality of oilseed crops for Europe. Euphytica.

[89] Lu C., Napier J.A., Clemente T.E., Cahoon E.B. (2011). New frontiers in oilseed biotechnology: Meeting the global demand for vegetable oils for food, feed, biofuel, and industrial applications. Curr. Opin. Biotechnol..

[90] Salas H., Castillejos L., Ferret A. Camelina meal, camelina expeller and camelina hulls: Nutritional characterization and in vitro digestibility. Proceedings of the XVII Jornadas sobre Producción Animal.

[91] Puzio I., Graboś D., Bieńko M., Radzki R.P., Nowakiewicz A., Kosior-Korzecka U. (2021). Camelina Oil Supplementation Improves Bone Parameters in Ovariectomized Rats. Animals.

[92] Boyle C., Hansen L., Hinnenkamp C., Ismail B.P. (2018). Emerging camelina protein: Extraction, modification, and structural/functional characterization. J. Am. Oil Chem. Soc..

[93] Karvonen H.M., Aro A., Tapola N.S., Salminen I., Uusitupa M.I., Sarkkinen E.S. (2002). Effect of [alpha]-linolenic acid [ndash] rich *Camelina sativa* oil on serum fatty acid composition and serum lipids in hypercholesterolemic subjects. Metab. Clin. Exp..

[94] Pekel A., Kim J., Chapple C., Adeola O. (2015). Nutritional characteristics of camelina meal for 3-week-old broiler chickens. Poult. Sci..

[95] Cais-Sokolińska D., Majcher M., Pikul J., Bielińska S., Czauderna M., Wójtowski J. (2011). The effect of *Camelina sativa* cake diet supplementation on sensory and volatile profiles of ewe’s milk. Afr. J. Biotechnol..

[96] Szterk A., Roszko M., Sosińska E., Derewiaka D., Lewicki P. (2010). Chemical composition and oxidative stability of selected plant oils. J. Am. Oil Chem. Soc..

[97] Ratusz K., Symoniuk E., Wroniak M., Rudzińska M. (2018). Bioactive compounds, nutritional quality and oxidative stability of cold-pressed camelina (*Camelina sativa* L.) oils. Appl. Sci..

[98] Cieslak A., Stanisz M., Wojtowski J., Pers-Kamczyc E., Szczechowiak J., El-Sherbiny M., Szumacher-Strabel M. (2013). *Camelina sativa* affects the fatty acid contents in M. longissimus muscle of lambs. Eur. J. Lipid Sci. Technol..

[99] Moloney A., Woods V., Crowley J. (1998). A note on the nutritive value of camelina meal for beef cattle. Irish J. Agric. Food Res..

[100] Mihhejev K., Henno M., Ots M., Rihma E., Elias P., Kuusik S., Kärt O. (2007). Effects of fat-rich oil cakes on cheese fatty acid composition, and on cheese quality. Vet. Zootech..

[101] Maxin G., Ouellet D., Lapierre H. (2013). Ruminal degradability of dry matter, crude protein, and amino acids in soybean meal, canola meal, corn, and wheat dried distillers grains. J. Dairy Sci..

[102] Schulz F., Westreicher-Kristen E., Knappstein K., Molkentin J., Susenbeth A. (2018). Replacing maize silage plus soybean meal with red clover silage plus wheat in diets for lactating dairy cows. J. Dairy Sci..

[103] Paula E., Monteiro H., Silva L., Benedeti P., Daniel J., Shenkoru T., Broderick G., Faciola A. (2017). Effects of replacing soybean meal with canola meal differing in rumen-undegradable protein content on ruminal fermentation and gas production kinetics using 2 in vitro systems. J. Dairy Sci..

[104] Mjoun K., Kalscheur K., Hippen A., Schingoethe D. (2010). Ruminal degradability and intestinal digestibility of protein and amino acids in soybean and corn distillers grains products. J. Dairy Sci..

[105] Lamminen M., Halmemies-Beauchet-Filleau A., Kokkonen T., Vanhatalo A., Jaakkola S. (2019). The effect of partial substitution of rapeseed meal and faba beans by Spirulina platensis microalgae on milk production, nitrogen utilization, and amino acid metabolism of lactating dairy cows. J. Dairy Sci..

[106] Heuzé V., Tran G., Sauvant D., Lessire M., Lebas F. (2017). Rapeseed Meal. Feedipedia, a Programme by INRA, CIRAD, AFZ and FAO.

[107] Bora P. (2014). Anti-nutritional factors in foods and their effects. J. Acad. Ind. Res..

[108] Mzengereza K., Ishikawa M., Koshio S., Yokoyama S., Yukun Z., Shadrack R.S., Seo S., Kotani T., Dossou S., Basuini M.F.E. (2021). Growth Performance, Growth-Related Genes, Digestibility, Digestive Enzyme Activity, Immune and Stress Responses of de novo Camelina Meal in Diets of Red Seabream (*Pagrus major*). Animals.

[109] Sizmaz O., Calik A., Sizmaz S., Yildiz G. (2016). A comparison of camelina meal and soybean meal degradation during incubation with rumen fluid as tested in vitro. Ank. Univ. Vet. Fak. Derg..

[110] Matthäus B., Angelini L.G. (2005). Anti-nutritive constituents in oilseed crops from Italy. Ind. Crops Prod..

[111] Fahey J.W., Zalcmann A.T., Talalay P. (2001). The chemical diversity and distribution of glucosinolates and isothiocyanates among plants. Phytochemistry.

[112] Tripathi M., Mishra A. (2007). Glucosinolates in animal nutrition: A review. Anim. Feed Sci. Technol..

[113] Knutsen H.K., Alexander J., Barregård L., Bignami M., Brüschweiler B., Ceccatelli S., Dinovi M., Edler L., Grasl-Kraupp B., EFSA Panel on Contaminants in the Food Chain (CONTAM) (2016). Erucic acid in feed and food. EFSA J..

[114] Hrastar R., Abramovič H., Košir I.J. (2012). In situ quality evaluation of *Camelina sativa* landrace. Eur. J. Lipid Sci. Technol..

[115] Brandao V., Dai X., Paula E., Silva L., Marcondes M., Shenkoru T., Poulson S., Faciola A. (2018). Effect of replacing calcium salts of palm oil with camelina seed at 2 dietary ether extract levels on digestion, ruminal fermentation, and nutrient flow in a dual-flow continuous culture system. J. Dairy Sci..

[116] Pekel A., Horn N., Adeola O. (2017). The efficacy of dietary xylanase and phytase in broiler chickens fed expeller-extracted camelina meal. Poult. Sci..

[117] Rajapakse B. (2015). Nutritive evaluation of mechanically-pressed camelina (*Camelina sativa*), carinata (*Brassica carinata*) and soybean (*Glycine max*) meals for broiler chickens. Master’s Thesis.

[118] Hanschen F.S. (2020). Domestic boiling and salad preparation habits affect glucosinolate degradation in red cabbage (*Brassica oleracea* var. capitata f. rubra). Food Chem..

[119] Jing B., Guo R., Wang M., Zhang L., Yu X. (2020). Influence of seed roasting on the quality of glucosinolate content and flavor in virgin rapeseed oil. Lwt.

[120] Oerlemans K., Barrett D.M., Suades C.B., Verkerk R., Dekker M. (2006). Thermal degradation of glucosinolates in red cabbage. Food Chem..

[121] Fredlund K., Asp N.-G., Larsson M., Marklinder I., Sandberg A.-S. (1997). Phytate reduction in whole grains of wheat, rye, barley and oats after hydrothermal treatment. J. Cereal Sci..

[122] Mukhopadhyay N.A. (1999). Effect of fermentation on the nutritive value of sesame seed meal in the diets for rohu, Labeo rohita (Hamilton), fingerlings. Aquaculture Nutr..

[123] Zeb A., Bibi N., Badshah A., Ter Meulen U. (2006). Effect of dry and wet heat treatments on rapeseed phenolics. Adv. Food Sci..

[124] Stedman J., Hill R. (1987). Voluntary food intake in a limited time of lambs and calves given diets containing rapeseed meal from different types and varieties of rape, and rapeseed meal treated to reduce the glucosinolate concentration. Anim. Sci..

[125] Clements K., Gleeson V., Slaytor M. (1994). Short-chain fatty acid metabolism in temperate marine herbivorous fish. J. Comp. Physiol. B.

[126] Chiang G., Lu W., Piao X., Hu J., Gong L., Thacker P. (2009). Effects of feeding solid-state fermented rapeseed meal on performance, nutrient digestibility, intestinal ecology and intestinal morphology of broiler chickens. Asian-Australas. J. Anim. Sci..

[127] Ahmed A., Zulkifli I., Farjam A.S., Abdullah N., Liang J.B., Awad E.A. (2014). Effect of solid state fermentation on nutrient content and ileal amino acids digestibility of canola meal in broiler chickens. Ital. J. Anim. Sci..

[128] Heerd D., Yegin S., Tari C., Fernandez-Lahore M. (2012). Pectinase enzyme-complex production by Aspergillus spp. in solid-state fermentation: A comparative study. Food Bioprod. Process..

[129] Olukomaiya O.O. (2021). Utilization of Solid-State Fermented Canola Meal, Camelina Meal and Lupin as Potential Protein Sources in the Diets of Broiler Chickens. Ph.D. Thesis.

[130] Olukomaiya O., Fernando C., Mereddy R., Li X., Sultanbawa Y. Nutritional analysis of solid-state fermented canola meal (an improved protein source for broilers). Proceedings of the 22nd European Symposium on Poultry Nutrition.

[131] Olukomaiya O.O., Fernando C., Mereddy R., Li X., Sultanbawa Y. (2020). Nutritional composition of solid-state fermented camelina meal (an enriched protein source for broiler chickens). Multidiscip. Digit. Publ. Inst. Proc..

[132] Olukomaiya O.O., Adiamo O.Q., Fernando W.C., Mereddy R., Li X., Sultanbawa Y. (2020). Effect of solid-state fermentation on proximate composition, anti-nutritional factor, microbiological and functional properties of lupin flour. Food Chem..

[133] Thacker P., Widyaratne G. (2012). Effects of expeller pressed camelina meal and/or canola meal on digestibility, performance and fatty acid composition of broiler chickens fed wheat–soybean meal-based diets. Arch. Anim. Nutr..

[134] Demorest Z.L., Coffman A., Baltes N.J., Stoddard T.J., Clasen B.M., Luo S., Retterath A., Yabandith A., Gamo M.E., Bissen J. (2016). Direct stacking of sequence-specific nuclease-induced mutations to produce high oleic and low linolenic soybean oil. BMC Plant Biol..

[135] Haun W., Coffman A., Clasen B.M., Demorest Z.L., Lowy A., Ray E., Retterath A., Stoddard T., Juillerat A., Cedrone F. (2014). Improved soybean oil quality by targeted mutagenesis of the fatty acid desaturase 2 gene family. Plant Biotechnol. J..

[136] Lu C., Kang J. (2008). Generation of transgenic plants of a potential oilseed crop *Camelina sativa* by Agrobacterium-mediated transformation. Plant Cell Rep..

[137] Kang J., Snapp A.R., Lu C. (2011). Identification of three genes encoding microsomal oleate desaturases (FAD2) from the oilseed crop *Camelina sativa*. Plant Physiol. Biochem..

[138] Nguyen H.T., Silva J.E., Podicheti R., Macrander J., Yang W., Nazarenus T.J., Nam J.W., Jaworski J.G., Lu C., Scheffler B.E. (2013). Camelina seed transcriptome: A tool for meal and oil improvement and translational research. Plant Biotechnol. J..

[139] Morineau C., Bellec Y., Tellier F., Gissot L., Kelemen Z., Nogué F., Faure J.D. (2017). Selective gene dosage by CRISPR-Cas9 genome editing in hexaploid *Camelina sativa*. Plant Biotechnol. J..

[140] Jiang W.Z., Henry I.M., Lynagh P.G., Comai L., Cahoon E.B., Weeks D.P. (2017). Significant enhancement of fatty acid composition in seeds of the allohexaploid, *Camelina sativa*, using CRISPR/Cas9 gene editing. Plant Biotechnol. J..

[141] Ozseyhan M.E., Kang J., Mu X., Lu C. (2018). Mutagenesis of the FAE1 genes significantly changes fatty acid composition in seeds of *Camelina sativa*. Plant Physiol. Biochem..

[142] Huang A., Coutu C., Harrington M., Rozwadowski K., Hegedus D.D. (2021). Engineering a feedback inhibition-insensitive plant dihydrodipicolinate synthase to increase lysine content in *Camelina sativa* seeds. Transgenic Res..

[143] Lyzenga W.J., Harrington M., Bekkaoui D., Wigness M., Hegedus D.D., Rozwadowski K.L. (2019). CRISPR/Cas9 editing of three CRUCIFERIN C homoeologues alters the seed protein profile in *Camelina sativa*. BMC Plant Biol..

[144] Lock A.L., Shingfield K.J. (2004). Optimising milk composition. BSAP Occas. Publ..

[145] Harvatine K., Allen M. (2006). Effects of fatty acid supplements on feed intake, and feeding and chewing behavior of lactating dairy cows. J. Dairy Sci..

[146] Dai X., Weimer P.J., Dill-McFarland K.A., Brandao V.L., Suen G., Faciola A.P. (2017). Camelina seed supplementation at two dietary fat levels change ruminal bacterial community composition in a dual-flow continuous culture system. Front. Microbiol..

[147] Wachira A., Sinclair L., Wilkinson R., Hallett K., Enser M., Wood J. (2000). Rumen biohydrogenation of n-3 polyunsaturated fatty acids and their effects on microbial efficiency and nutrient digestibility in sheep. J. Agric. Sci..

[148] Chilliard Y., Glasser F., Ferlay A., Bernard L., Rouel J., Doreau M. (2007). Diet, rumen biohydrogenation and nutritional quality of cow and goat milk fat. Eur. J. Lipid Sci. Technol..

[149] Shingfield K., Bonnet M., Scollan N. (2013). Recent developments in altering the fatty acid composition of ruminant-derived foods. Animal.

[150] Destaillats F., Wolff R.L., Precht D., Molkentin J. (2000). Study of individual trans-and cis-16: 1 isomers in cow, goat, and ewe cheese fats by gas-liquid chromatography with emphasis on the trans-Δ3 isomer. Lipids.

[151] Harfoot C.G., Christie W.W. (1981). Lipid metabolism in the rumen. Lipid Metabolism in Ruminant Animals.

